# An evolutionary game perspective on quantised consensus in opinion dynamics

**DOI:** 10.1371/journal.pone.0209212

**Published:** 2019-01-04

**Authors:** Michalis Smyrnakis, Dario Bauso, Tembine Hamidou

**Affiliations:** 1 Learning and Game Theory Laboratory, New York University Abu Dhabi,Abu Dhabi United Arab Emirates; 2 Jan C. Willems Center for Systems and Control, ENTEG, Fac. Science and Engineering, University of Groningen, Groningen, Netherlands; 3 Dip. dell’Innovazione Industriale e Digitale (DIID), Università di Palermo, Palermo, Italy; University of Zaragoza, SPAIN

## Abstract

Quantised consensus has been used in the context of opinion dynamics. In this context agents interact with their neighbours and they change their opinion according to their interests and the opinions of their neighbours. We consider various quantised consensus models, where agents have different levels of susceptibility to the inputs received from their neighbours. The provided models share similarities with collective decision making models inspired by honeybees and evolutionary games. As first contribution, we develop an evolutionary game-theoretic model that accommodates the different consensus dynamics in a unified framework. As second contribution, we study equilibrium points and extend such study to the symmetric case where the transition probabilities of the evolutionary game dynamics are symmetric. Symmetry is associated with the case of equally favourable options. As third contribution, we study stability of the equilibrium points for the different cases. We corroborate the theoretical results with some simulations to study the outcomes of the various models.

## 1 Introduction

Multi-agent systems find numerous applications in various research areas. Agents interact and make decisions according to their selfish interests and the behaviour of the other agents. A topic of increasing interest in various research areas is the consensus problem. In this problem, agents are represented as nodes of a graph, directed or undirected, and the existence of an edge between two nodes denotes the ability of two agents to communicate. Then the goal is for the nodes to seek agreement on a value of a common quantity or variable. These variables include but are not limited to resources which agents want to share, their cooperation levels and communication bandwidth [1, 2].

In this article we are interested in consensus problems where the decision variables of the agents are discrete, i.e. their choices are integer numbers. In engineering sciences the consensus problem when the decision variable is discrete is often called “quantised consensus”. It can emerge due to: constraints in communications, bounded capacity of the memory of sensors and noisy measurements and discrete nature of the decision variables [1, 3–6]. Another research area that considers discrete consensus variables is opinion dynamics [7–17].

The opinion of each agent is represented by an integer and the interacting agents can change their opinions based on the input they receive from neighbors [18–20]. Henceforth, the terms opinion and decision variable will be used interchangeably.

Under a macroscopic representation of the quantised consensus problem, agents interact with their neighbours and change their actions with a certain probability. Depending on the characteristics of the agents, i.e. crowd seeking or crowd adverse behaviour, these probabilities depend on the number of agents selecting each decision variable.

The main contributions of this paper are as follows. Firstly, a microscopic model, the quantised consensus process, is considered. In this process agents are able to choose among three possible options. A game is then developed which is equivalent to the consensus process. The game is an evolutionary one with three available actions per player and describes the evolution of the population from a macroscopic perspective. This game can be seen as an evolutionary version of a two player strategic form game. Each player can be in one of three possible states, namely *coordinators*, *defectors* and *neutrals*. The developed evolutionary game builds on the notion of expected gain pairwise comparison which was first proposed in [21]. The relevance of such a result is that we bring in a unified framework, namely the evolutionary game, five different consensus dynamics, which we refer to as Case 1 to 5. These cases model the impact of other agents’ opinions to a single individual’s opinion through different reward functions. Each reward function corresponds therefore to a different quantised consensus problem.

As second contribution, the proposed game is cast as a Markov process and the equilibrium points are investigated through the analysis of the Markov chain. We obtain that the three vertices of the simplex in $R_{3}$ are all equilibrium points. These vertices correspond to the cases where all the agents converge to the same option or remain all uncommitted. A fourth equilibrium point may be obtained which lies in the interior of the simplex and where the populations committed to either one option or the other are related by a proportionality linear rule. We study such an equilibrium point in the symmetric case where the transition probabilities from and to the uncommitted state are symmetric.

As third contribution, we provide a stability analysis of the equilibrium points for each case. Different stability properties are obtained depending on the agents’ behaviour and their tendency to follow their peers.

The rest of this paper is organised as follows. In Section 2, relevant work is provided. In Section 3, we formulate the problem, and we introduce the corresponding game formulation. The unified framework between the consensus models and the Markov processes which emanate from the game theoretic formulation is also presented in this section. In Section 4, the analysis of five different models which correspond to different agents behaviours is presented. In Section 5, theoretical analysis of the proposed models and simulation results are presented. Finally Section 6 contains a discussion on our findings and directions for future works.

## 2 Related Work

Consensus algorithms are considered as the canonical example when coordination mechanisms in multi-agent systems are considered. Agents which use consensus algorithms aim to reach agreement when the common value of interest is considered. This is achieved by taking into account the pairwise interactions between agents. These interactions then are analysed using consensus algorithms. Consensus algorithms have been used in order to find solutions among others in wireless networks [22], distributed multi-agent optimization [23, 24], signal processing [25], numerical estimation [26, 27] and opinion dynamics [20, 28].

Various consensus algorithms have been proposed in the literature with a variety of features studied depending on the research area they were introduced. In [29] a literature review of opinion dynamics models is presented. The reviewed algorithms were classified in two categories depending on the usage or not of external information. Additionally in each category the algorithms were separated to discrete and continuous depending on the form of the opinion they use. In [30] another survey of various opinion dynamics is presented. In this survey opinion dynamics are considered as a fusion process of individual opinions.

In [31] a Markov model for disease spreading is presented, after a brief literature review of various epidemic and rumour spreading algorithms. In contrast to the proposed methodology, in [31] constant transition probabilities were used in the Markov model.

In [32] the consensus of societies towards social norms were studied through evolutionary games. The players of the game were penalised if they were observed to deviate from the norm. In [33] various models of the influence of other agents’ opinions on an agent’s decision were studied. An approach which considers local information in the consensus problem was proposed in [34].

The interconnection between consensus and distributed optimisation was studied in [23, 35]. The impact of different media and their particular size in shaping of an opinion is presented in [36].

In [37, 38] the convergence properties and the speed of convergence of gossip algorithms have been studied for various network topologies. In these works, in contrast to this article, continuous decision variables have been used by the agents, which lead to a different update rule for the consensus algorithm. Additionally in the majority of the gossip algorithms an aggregation of the opinions of each neighbour leads to a change to an agent’s opinion. In contrast, in this article since the decision variables are discrete the agents are influenced by the popularity of that particular opinion according to their characteristics.

A bio-inspired methodology which can be used to model consensus of interacting agents comes from bee colonies. Particularly, from the method which bees adopt in order to choose their nesting site. Scouts are sent to potential places for nesting and depending on the suitability of each place the scouts persuade, or “recruit”, the other uncommitted members by performing a waggle dance. Additionally, in order to stop other scouts from recruiting more uncommitted members, scouts committed to one option try to intercept the waggle dance of scouts committed to a different option. This can be viewed as a form of cross-inhibitory signal.

In analogy with the agent based decision making; the waggle dance represents agents who intend to influence other agents to be committed to the same opinion as the one they have. The cross-inhibitory signal on the other hand are agents who attempt to persuade an agent to a different opinion than the one it currently has. Alternatively, consider the case where the formation of an opinion is a process with two parts. The first one is the influence of an agent’s peers with the same opinion, which we assimilate to the waggle dance. The second one is the influence of agent’s peers which have different opinion than his current one.

Using this formulation behaviours as opinionated agents and crowd seeking or crowd adverse agents can be modelled. In particular, when the cross inhibitory signals are considered, the degree of an opinionated individual can be modelled. An opinionated individual will need more of his peers in order to change his opinion towards another one, than a less opinionated one. When the waggle dance is considered a crowd seeking agent would choose the action which is followed by the majority of his peers, and the opposite will happen for a crowd averse agent.

## 3 Generic problem formulation

In this article the influence that opinions of neighbouring agents have in the formation of an agent’s opinion is modelled. The underlying assumption is that the agents are able to choose one of three possible states to be, denoted by *X*, *Y* and *Z* hereafter. Each state will correspond to one of the possible options: “committed to opinion *X*”, “committed to opinion *Y*”, or not committed to any opinion (“committed to opinion *Z*”). Agents can change their opinion from *X* to *Z* or from *Y* to *Z* and from *Z* to *X* and *Y*.

### 3.1 Quantised consensus approach

Let the state of the reference agent at time *t* ≥ 0, which is henceforth referred to as agent *i*, be indicated by the variable $w_{t}^{i}\in \left\{X,Y,Z\right\}$.

This decision process can be cast as a quantised consensus problem. Consider the case of a well mixed population of N agents represented through a connected undirected graph G(N,E). Each agent is represented as a node of the graph and an edge connects two nodes if agents can interact, i.e. they are neighbours. Let *w* be the vector of the discretised decision variables of all agents, we will write $w_{t}^{i}=w$ to indicate that agent *i*’s decision at time *t* is *w* and write $w_{t+1}^{i}\left(w_{t}^{i}=w\right)$ to denote the decision variable of the reference agent *i* at time *t* + 1 given that his value at time *t* was *w*, *w* ∈ {*X*, *Y*, *Z*}. For convenience of notation in the rest of the paper, if not otherwise stated, a variable without a time index will denote the variable at time *t*.

The evolution of the agents’ decisions can be illustrated by the following generic quantised consensus process, where $p_{\tilde{w}}$ is the probability of transitioning if the generic set of rules A are satisfied:

$$\begin{array}{c} w_{t+1}^{i}\left(w_{t}^{i}=w\right)=\{\begin{array}{cc} \tilde{w},\forall \;\tilde{w}\in \left\{X,Y,Z\right\},\;\tilde{w}\neq w & \text{with}\;\text{probabilility}\;p_{\tilde{w}}\;\text{under}\;a\;\text{set}\;\text{of}\;\text{rules}\;A,\\ w & \text{otherwise}. \end{array} \end{array}$$ (1)

Both the probabilities $p_{\tilde{w}}$ and the set of rules A will be introduced in the following sections, distinguishing five different cases.

To each microscopic dynamics we will associate a Markov process representation which describes the probability distribution of $w_{t}^{i}$ over the set {*X*, *Y*, *Z*}. In generic terms, the Markov process model can be expressed as

$$\begin{array}{c} \left[x_{t+1}\;y_{t+1}\;z_{t+1}\right]=\underset{=:P}{\underset{︸}{\left[\begin{array}{ccc} P_{XX} & P_{XY} & P_{XZ}\\ P_{YX} & P_{YY} & P_{YZ}\\ P_{ZX} & P_{ZY} & P_{ZZ} \end{array}\right]}}\left[x_{t}\;y_{t}\;z_{t}\right]. \end{array}$$ (2)

### 3.2 A unified framework based on evolutionary game-theoretic formulation

The aforementioned quantised consensus model is formulated from a microscopic perspective which looks at a single agent, who was referred to as the reference agent *i*. From a macroscopic perspective, which considers the evolution of the population over the three opinions, the different consensus dynamics can be cast as an evolutionary game. The relevance of such evolutionary game model is that it provides a unified modelling framework accommodating the five different opinion dynamics.

Before introducing the game-theoretic formulation, let us start by noting that the population dynamics can be described via a Markov process in terms of *x* and *y* since *x* + *y* + *z* = 1. The equations of the system’s dynamics in discrete-time are:

$$\begin{array}{c} \begin{array}{c} x_{t+1}=x_{t}-p_{XZ}x_{t}+p_{ZX}\left(1-x_{t}-y_{t}\right),\\ y_{t+1}=y_{t}-p_{YZ}y_{t}+p_{ZY}\left(1-x_{t}-y_{t}\right),\\ z_{t+1}=\left(1-x_{t}-y_{t}\right)-\left(p_{ZX}+p_{ZY}\right)\left(1-x_{t}-y_{t}\right)+p_{XZ}x_{t}+p_{YZ}y_{t}. \end{array} \end{array}$$ (3)

The third equation of (3) can be written as *z*^*t*+1^ = 1 − *x*^*t*+1^ − *y*^*t*+1^. Therefore (3) reduces to:

$$\begin{array}{c} \begin{array}{c} x_{t+1}=x_{t}-p_{XZ}x_{t}+p_{ZX}\left(1-x_{t}-y_{t}\right),\\ y_{t+1}=y_{t}-p_{YZ}y_{t}+p_{ZY}\left(1-x_{t}-y_{t}\right).\; \end{array} \end{array}$$ (4)

To introduce the evolutionary game model, consider an identical payoff three action game which is played over a population of N individuals. Each player in this game chooses an action proportionally to the expected gain pairwise comparison in accordance with the definition provided in [21] which we copy and adapt below. The resulting evolutionary dynamics adds to the ones surveyed in [39]. In particular we have the following definition for the expected gain, which can be viewed as the fitness function of a player.

**Definition 1**. *For a generic n* × *n pay-off matrix, A the expected gain of action i when the current action of a player is j is defined as*:

$$\begin{array}{c} E_{ij}=\sum_{k=1}^{k=n}I\left(a_{ik}-a_{jk}\right)x_{k}, \end{array}$$ (5)

*where n is the number of available actions to the players, a*_*ik*_ *is the ik*_*th*_ *element of matrix A, x*_*k*_ *is the fraction of players that have chosen action k and* $I=\{\begin{array}{cc} a_{ik}-a_{jk} & if\;a_{ik}-a_{jk}>0\\ 0 & otherwise \end{array}.$

The agents are now viewed as *players* and their opinions are referred to as *actions*. Let us also denote the fraction of the population who are in state *X* as *x*, the fraction of the population who are not committed (state *Z*) as *z* and the fraction of the population who are in state *Y* as *y*. This is equivalent to the portion of agents whose opinions are *w*^*i*^ = *X*, *w*^*i*^ = *Z* and *w*^*i*^ = *Y* respectively. Since the population is constant the three fractions sum up to one, i.e. *x* + *y* + *z* = 1.

Given that transitions from *X* to *Y* are not allowed this should be also reflected in the pay-off matrix by setting the rewards of these transitions to zero. Additionally, the rewards should also reflect the tendency of the players to choose similar actions to other players and therefore penalise deviations from others. We will briefly refer to such a phenomenon as *crowd-seeking behaviour*. By taking this into account and considering that *x* + *y* + *z* = 1, the following pay-off matrix can be considered:

$$A\left(X,Y\right)=\begin{array}{l} X\\ Y\\ Z \end{array}(\begin{array}{ccc} X & Y & Z\\ a_{11}f_{1}\left(\cdot \right) & -a_{12}f_{2}\left(\cdot \right) & 0\\ -a_{21}f_{3}\left(\cdot \right) & a_{22}f_{4}\left(\cdot \right) & 0\\ 0 & 0 & 0 \end{array}),$$ (6)

where *f*_*i*_(⋅) *i* = 1,…, 4 are arbitrary functions of *x* and *y* which will be defined later to accommodate the five different opinion dynamics. From (5), the transition probabilities between actions are given as follows:

$$\begin{array}{c} \begin{array}{cc} p_{ZX}=a_{11}xf_{1}\left(\cdot \right), & \;p_{XZ}=a_{12}yf_{2}\left(\cdot \right),\\ p_{YZ}=a_{21}xf_{3}\left(\cdot \right), & \;p_{ZY}=a_{22}yf_{4}\left(\cdot \right). \end{array} \end{array}$$ (7)

Substituting the above transition probabilities in (4) we obtain:

$$\begin{array}{c} \begin{array}{c} x_{t+1}=x_{t}-a_{12}f_{2}\left(\cdot \right)x_{t}y_{t}+a_{11}x_{t}f_{1}\left(\cdot \right)\left(1-x_{t}-y_{t}\right),\\ y_{t+1}=y_{t}-a_{21}f_{3}\left(\cdot \right)x_{t}y_{t}+a_{22}y_{t}f_{4}\left(\cdot \right)\left(1-x_{t}-y_{t}\right),\; \end{array} \end{array}$$ (8)

which is the dynamics we analyse in the rest of this paper.

**Fig 1:**
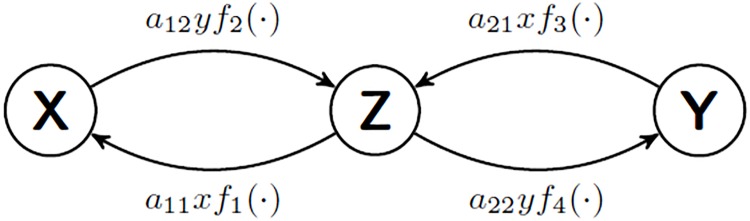
Markov chain emerging from the generic form of A.

The resulting evolution of *x*, *y* and *z* are described by the Markov process which is depicted in Fig 1.

## 4 Analysis of particular models

In this section various opinion dynamics mechanisms are presented. Each of them represents a different kind of influence that the neighbours of an agent have in his opinion formation process. These models are build on different assumptions about how many of peers are needed to influence an agent to adopt a specific opinion. Following the jargon of bio-inspired collective decision making, the five cases considered in this article can be catalogued in terms of strength of the cross-inhibitory and the waggle dance signals. More specifically, in Case 1 both the cross-inhibitory and the waggle dance signals are linear. These can be considered as “broad minded” agents since they can reconsider their decisions by taking into account only the state of a single neighbour.

In Case 2 one observes a weak cross-inhibitory and a strong waggle dance signal. This second case deals with stubborn agents who compare the decisions of more than one of their neighbours in order to change their opinion with a given probability.

In Case 3 we have a strong cross-inhibitory and a weak waggle dance signal. The third case deals with stubbornness of uncommitted agents. Consider a reference agent who is not committed, i.e. his opinion is *w*^*i*^ = *Z*. He commits to opinion *X* or *Y* only if *m* randomly chosen neighbours have opinion *X* or *Y*, respectively. Committed agents use the opinion of a single randomly chosen neighbour.

Cases 4 and 5 are similar to Case 1. The difference is that the probabilities of changing opinion depend solely on the percentage of the peers that belong to one of the two opinions *X* and *Y* denoted by $\tilde{x}$ and $\tilde{y}$ respectively.

**Fig 2:**
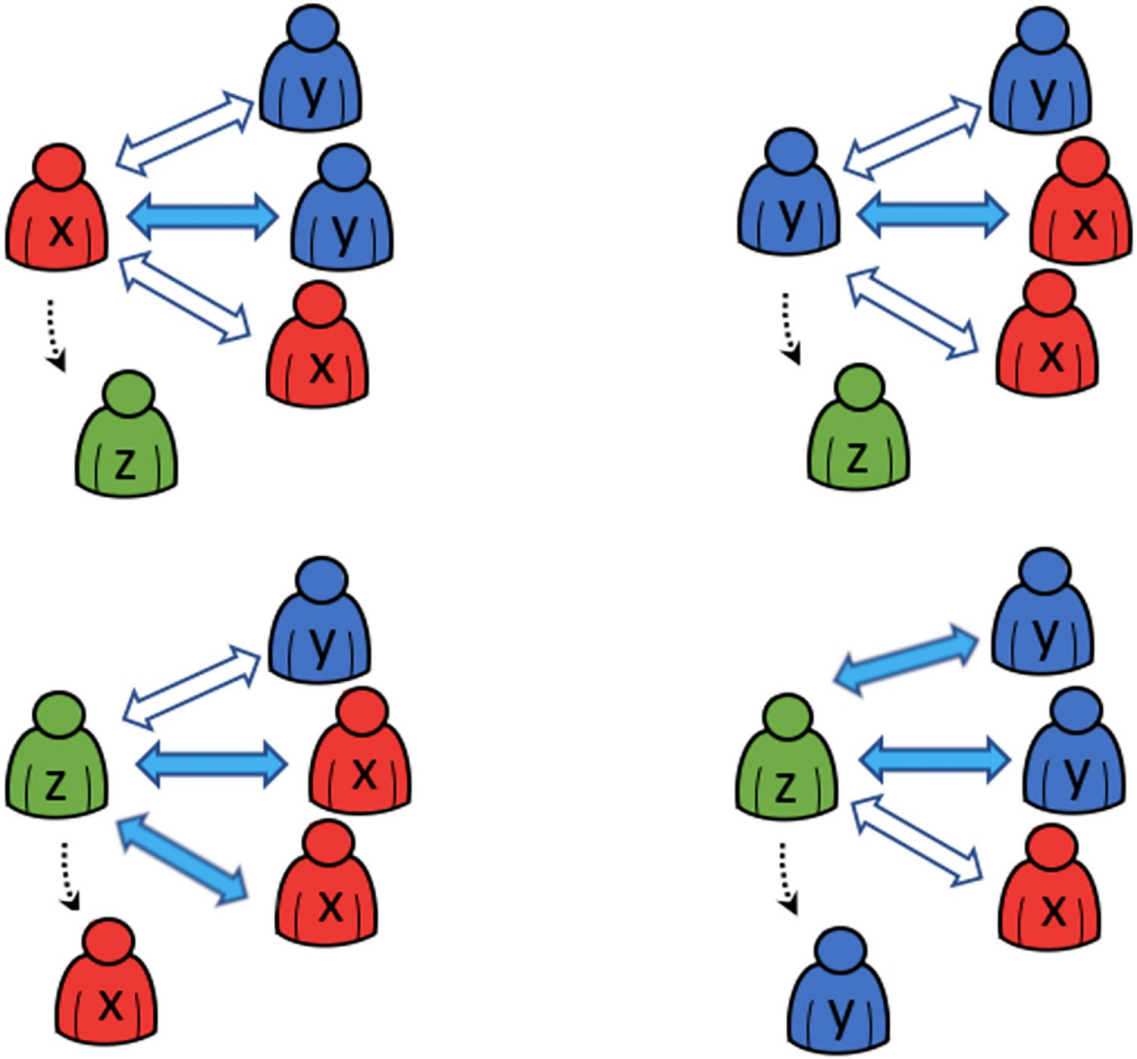
A generic quantised consensus process. Individual *X* meets *m* Individuals *Y* and mutates into Individual *Z* with probability *P*_*XZ*_ = *p*_1_ (top-left); Individual *Y* meets *m* Individuals *X* and mutates into Individual *Z* with probability *P*_*YZ*_ = *p*_2_ (top-right); Individual *Z* meets *d* Individuals *X* and mutates into Individual *X* with probability *P*_*ZX*_ = *p*_3_ (bottom-left); Individual *Z* meets *d* Individuals *Y* and mutates into Individual *Y* with probability *P*_*ZY*_ = *p*_4_ (bottom-right).

Let $M_{t}$ and $D_{t}$ denote two sets of *m* and *d* randomly selected neighbours of the reference agent *i* at time *t*. The generic form of the quantised consensus process for a set of decision rules A, which emanate from the aforementioned five cases, is illustrated in Fig 2 and is defined as:

$$\begin{array}{c} w_{t+1}^{i}\left(w_{t}^{i}=X\right)=\{\begin{array}{cc} Z & \text{with}\;\text{probabilility}\;p_{1},\;\text{if}\;w_{t}^{j}=Y,\;\forall j\in M_{t}\\ X & \text{otherwise}; \end{array} \end{array}$$ (9)

$$\begin{array}{c} w_{t+1}^{i}\left(w_{t}^{i}=Y\right)=\{\begin{array}{cc} Z & \text{with}\;\text{probabilility}\;p_{2},\;\text{if}\;w_{t}^{j}=X,\;\forall j\in M_{t},\\ Y & \text{otherwise}; \end{array} \end{array}$$ (10)

$$\begin{array}{c} w_{t+1}^{i}\left(w_{t}^{i}=Z\right)=\{\begin{array}{cc} X & \text{with}\;\text{probabilility}\;p_{3},\;\text{if}\;w_{t}^{j}=X,\;\forall j\in D_{t},\\ Y & \text{with}\;\text{probabilility}\;p_{4},\;\text{if}\;w_{t}^{j}=Y,\;\forall j\in D_{t},\\ Z & \text{otherwise}. \end{array} \end{array}$$ (11)

The cardinality of the sets M and D changes according to each case and are summarised in Table 1.

**Table 1:** Cardinality of neighbour sets and probabilities of changing opinion for each case.

	|M|	|D|	*p*_1_	*p*_2_	*p*_3_	*p*_4_
Case 1	1	1	*p*_1_	*p*_2_	*p*_3_	*p*_4_
Case 2	*m*	1	*p*_1_	*p*_2_	*p*_3_	*p*_4_
Case 3	1	d	*p*_1_	*p*_2_	*p*_3_	*p*_4_
Case 4	1	1	$p_{1}\frac{1}{\tilde{x}+\tilde{y}}$	$p_{2}\frac{1}{\tilde{x}+\tilde{y}}$	*p*_3_	*p*_4_
Case 4	1	1	*p*_1_	*p*_2_	$p_{3}\frac{1}{\tilde{x}+\tilde{y}}$	$p_{4}\frac{1}{\tilde{x}+\tilde{y}}$

The corresponding transition matrix *P* of the Markov process can be defined as:

$$\begin{array}{c} P=[\begin{array}{ccc} \left(1-p_{1}\right)I_{My}+\left(1-I_{My}\right) & 0 & p_{1}I_{My}\\ 0 & \left(1-p_{2}\right)I_{Mx}+\left(1-I_{Mx}\right) & p_{2}I_{Mx}\\ p_{3}I_{Dx} & p_{4}I_{Dy} & \left(1-p_{3}\right)I_{Dx}+\left(1-p_{4}\right)I_{Dy}+{\tilde{I}}_{Dxy} \end{array}]. \end{array}$$ (12)

where *I*_*Mx*_ and *I*_*My*_, *I*_*Dx*_ and *I*_*Dy*_ are defined as:

$$\begin{array}{c} I_{Mx}=\{\begin{array}{cc} 1 & \text{if}\;w_{t}^{j}=X\text{,}\;\;\;\forall j\in M_{t},\\ 0 & \text{otherwise,} \end{array}\;I_{My}=\{\begin{array}{cc} 1 & \text{if}\;w_{t}^{j}=Y\text{,}\;\;\;\forall j\in M_{t},\\ 0 & \text{otherwise,} \end{array} \end{array}$$

$$\begin{array}{c} I_{Dx}=\{\begin{array}{cc} 1 & \text{if}\;w_{t}^{j}=X\text{,}\;\;\;\forall j\in D_{t},\\ 0 & \text{otherwise,} \end{array}\;I_{My}=\{\begin{array}{cc} 1 & \text{if}\;w_{t}^{j}=Y\text{,}\;\;\;\forall j\in D_{t},\\ 0 & \text{otherwise.} \end{array} \end{array}$$

and ${\tilde{I}}_{Dxy}=1-I_{Mx}-I_{My}.$

Analytical definitions of the consensus process and their corresponding transition probabilities for each case are provided in S1 File.

## 5 Results

In this section theoretical and simulation results are presented for various opinion dynamics models.

### 5.1 Theoretical results

We are in the position to establish the first main result that states that the five opinion dynamics can be formulated in a unified framework as evolutionary game dynamics for different choices of the functions *f*_*i*_(⋅), *i* = 1,…, 4.

**Theorem 1**. *The evolutionary game* (8) *describes the population dynamics in Cases 1 to 5 for the following choices of functions f*_*i*_(.), *i* = 1,…, 4:

$$\begin{array}{c} \begin{array}{ccccc} \left(Case\;1\right) & \;f_{1}\left(\cdot \right)=1, & f_{2}\left(\cdot \right)=1, & f_{3}\left(\cdot \right)=1, & f_{4}\left(\cdot \right)=1,\\ \left(Case\;2\right) & \;f_{1}\left(\cdot \right)=1, & f_{2}\left(\cdot \right)=y^{m-1}, & f_{3}\left(\cdot \right)=x^{m-1}, & f_{4}\left(\cdot \right)=1,\\ \left(Case\;3\right) & \;f_{1}\left(\cdot \right)=x^{m-1}, & f_{2}\left(\cdot \right)=1, & f_{3}\left(\cdot \right)=1, & f_{4}\left(\cdot \right)=y^{m-1},\\ \left(Case\;4\right) & \;f_{1}\left(\cdot \right)=1, & f_{2}\left(\cdot \right)=\frac{1}{x+y}, & f_{3}\left(\cdot \right)=\frac{1}{x+y}, & f_{4}\left(\cdot \right)=1,\\ \left(Case\;5\right) & \;f_{1}\left(\cdot \right)=\frac{1}{x+y}, & f_{2}\left(\cdot \right)=1, & f_{3}\left(\cdot \right)=1, & f_{4}\left(\cdot \right)=\frac{1}{x+y}. \end{array} \end{array}$$ (13)

The proof of Theorem 1 is provided in S2 File.

The equilibrium points of the various models of the previous section was also studied. From (8), the equilibrium points are obtained by equalising *x*_*t*+1_ = *x*_*t*_ and *y*_*t*+1_ = *y*_*t*_ which yields

$$\begin{array}{c} \begin{array}{c} \frac{P_{XZ}}{P_{ZX}}=\frac{a_{12}f_{2}\left(\cdot \right)x_{t}y_{t}}{a_{11}x_{t}f_{1}\left(\cdot \right)}=\left(1-x_{t}-y_{t}\right),\\ \frac{P_{YZ}}{P_{ZY}}=\frac{a_{21}f_{3}\left(\cdot \right)x_{t}y_{t}}{a_{22}y_{t}f_{4}\left(\cdot \right)}=\left(1-x_{t}-y_{t}\right).\; \end{array} \end{array}$$ (14)

The above can equivalently be written as

$$\begin{array}{c} \begin{array}{c} a_{12}f_{2}\left(\cdot \right)x_{t}y_{t}=a_{11}x_{t}f_{1}\left(\cdot \right)\left(1-x_{t}-y_{t}\right),\\ a_{21}f_{3}\left(\cdot \right)x_{t}y_{t}=a_{22}y_{t}f_{4}\left(\cdot \right)\left(1-x_{t}-y_{t}\right).\; \end{array} \end{array}$$ (15)

which implies:

$$\begin{array}{c} \begin{array}{c} y_{t}a_{12}f_{2}\left(\cdot \right)a_{22}f_{4}\left(\cdot \right)=x_{t}a_{11}f_{1}\left(\cdot \right)a_{21}f_{3}\left(\cdot \right). \end{array} \end{array}$$ (16)

In the next theorem we show that the vertices of the simplex in $R_{3}$ are equilibrium points and that there exists a fourth equilibrium point which satisfies the linear condition *y* = *qx* for given scalar *q* ≥ 0.

**Theorem 2**. *The following tuples are equilibrium points for the evolutionary game* (8):

(x=1,y=0,z=0),(x=0,y=1,z=0),(x=0,y=0,z=1).

*In addition, a fourth equilibrium point may exist of type* (*x*, *qx*, 1 − (1 + *q*)*x*).

The analytical form of the equilibrium types for the particular cases and the proof of Theorem 2 are provided in S3 File.

In the above theorem, the equilibrium point *y* = *qx* may be outside the simplex in $R_{3}$ which would make it not feasible. In the following, we investigate the conditions of feasibility in the case of symmetric parameters where *q* ≃ 1.

A list of the equilibrium points of the form *y* = *qx* and their corresponding feasibility conditions, in the symmetric case where *q* ≃ 1 are provided in the corollary in S4 File.

A way to study the stability of the equilibrium solutions is to study the eigenvalues of the Jacobian matrix *J* of the state space non-linear model, for each case, evaluated in each equilibrium point. Let λ = {λ_1_, λ_2_} be the eigenvalues of the Jacobian, then if evaluated in a specific solution |λ_*i*_| < 1, *i* ∈ {1, 2} this solution is stable. We are ready to establish the following stability properties.

**Theorem 3**. *Depending on the case considered the vertices of the simplex and the tuple* (*x*, *qx*, 1 − (1 + *q*)*x*) *can be stable equilibria*.

The form of the equilibria and proof of Theorem 3 for each case are provided in S5 File.

### 5.2 Simulations

**Fig 3:**
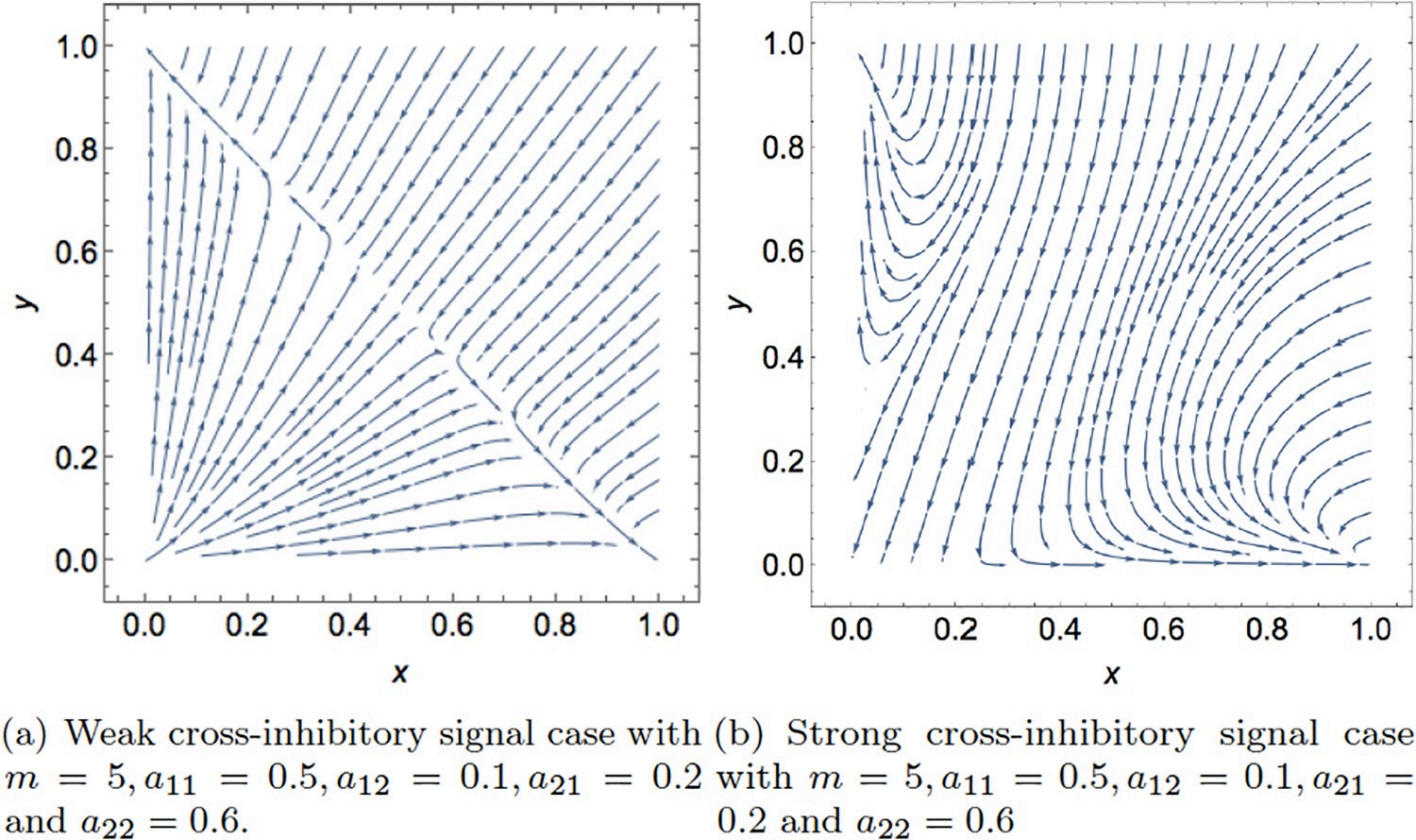
Phase portraits of the Markov decision processes.

In this section we provide some simulations to corroborate the theoretical results of the previous sections. Examples of the dynamics for the weak and strong cross inhibitory signal cases are depicted in Fig 3.

The interconnection between the quantised consensus models and their corresponding Markov process is shown for various reward matrices and uniformly chosen initial conditions. Random graphs, “Erdős-Rényi” networks [40], were used for the set-up of the quantised consensus formulation. In particular a graph G(N,E) with 1000 agents was generated, and the neighbours of each agent were uniformly chosen among the available N-1 agents with probability *p* = 0.2.

When a random graph is generated there is no guarantee of the minimum number of neighbours that agents will have. Some of the quantised consensus models of this article require that each agent will have at least *m* neighbours. For this reason in each simulation instance when a graph was generated, if the number of neighbours of any agent was less than *m* it was discarded. The process was repeated until a graph is generated with all nodes having at least *m* neighbours.

The initial values of the decision variable *w*_0_ were uniformly chosen. Then based on the same *w*_0_ and G(N,E) the five quantised consensus processes were used as coordination mechanism among the agents. The distribution of the agents among the three categories in each time step is reported.

The analysis of the evolution of the players’ behaviour, when the Markov process is considered, is indifferent to the structure of G(N,E). Therefore for the initialisation of the five Markov processes only the proportion of cooperators, defectors and neutrals in *w*_0_ was needed.

Four instances of the aforementioned process were considered. Each of them for different constant values (*a*_11_, *a*_12_, *a*_21_, *a*_22_). The constants which were used in each simulation instance are reported below.

$$\begin{array}{c} (\begin{array}{c} 0.2\\ 0.2\\ 0.2\\ 0.2 \end{array}), \end{array}$$ (17)

$$\begin{array}{c} (\begin{array}{c} 0.9\\ 0.2\\ 0.2\\ 0.2 \end{array}), \end{array}$$ (18)

$$\begin{array}{c} (\begin{array}{c} 0.9\\ 0.4\\ 0.4\\ 0.9 \end{array}), \end{array}$$ (19)

$$\begin{array}{c} (\begin{array}{c} 0.1\\ 0.5\\ 0.3\\ 0.6 \end{array}). \end{array}$$ (20)

**Fig 4:**
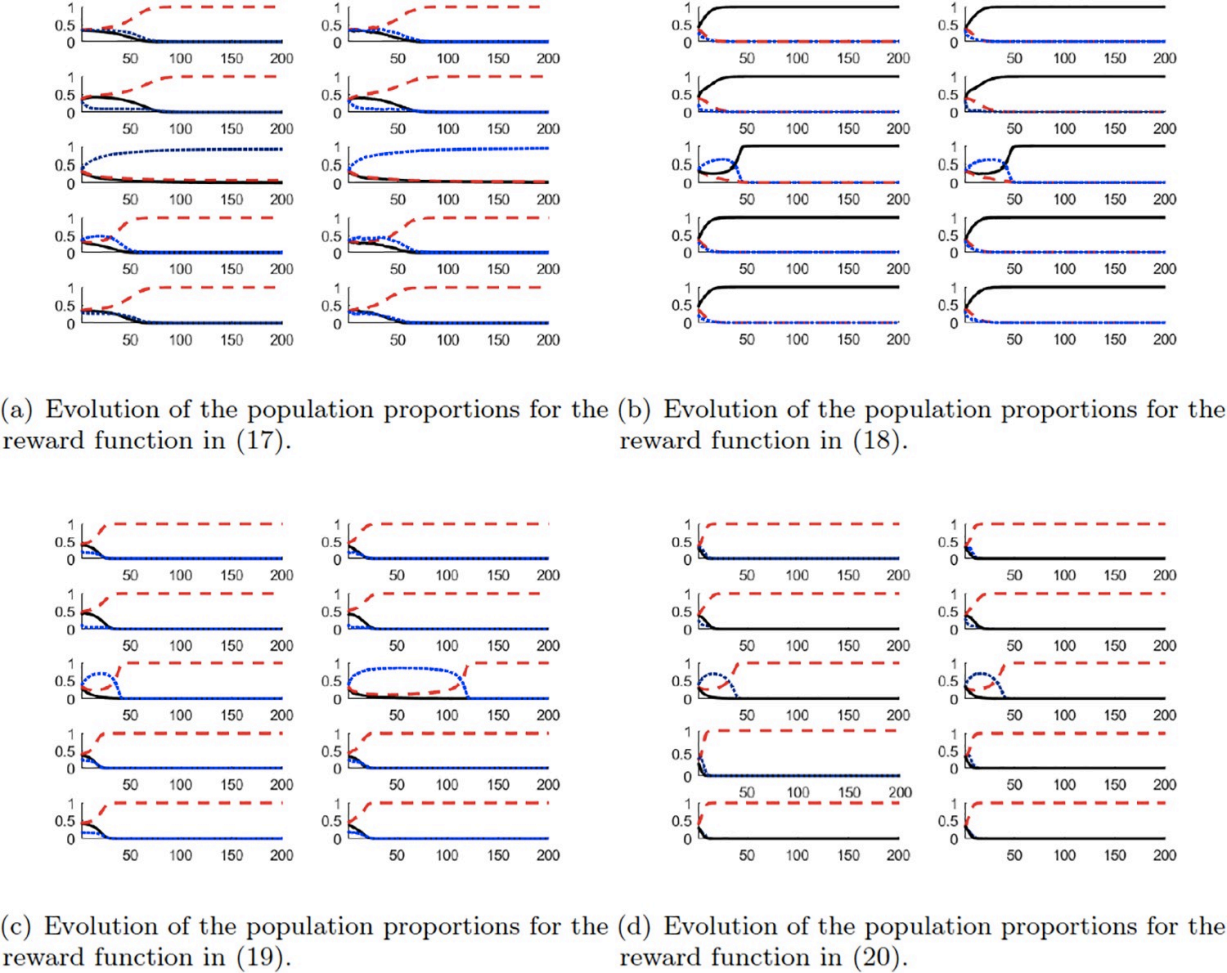
Simulation results. The players in *X*, *Y* and *Z*, are depicted as straiht, dashed and dotted lines in the figures. Each row represent one of the five cases. The left column corresponds to the results of the Markov process and the right column to the quantised consensus process. The *x*-axis corresponds to the iteration number and the *y*-axis to the distribution of the population over the three states.

The results of all four initial conditions are depicted in Fig 4. The results presented are for 200 iterations of both processes, consensus and Markov chains.

In all figures the quantised consensus and the Markov processes produce similar results.

**Fig 5:**
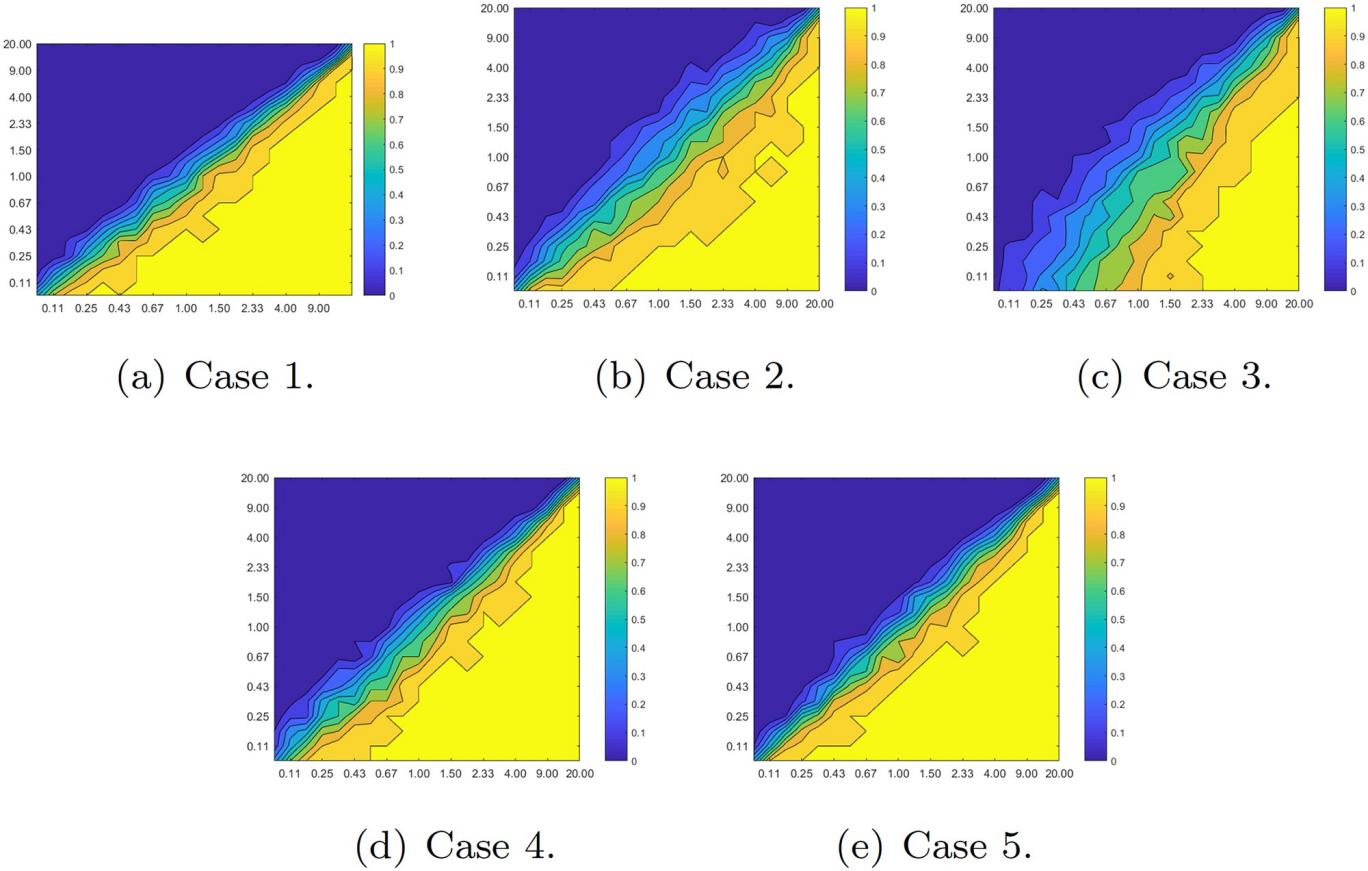
Convergence of the quantised consensus algorithm to the coordinators class with respect to the ratios $\frac{a_{12}}{a_{11}}$ and $\frac{a_{21}}{a_{22}}$. The x-axis represents the ratio $\frac{a_{12}}{a_{11}}$ and the y-axis the ratio $\frac{a_{21}}{a_{22}}$.

The effect of the reward matrix, and in particular the impact of the ratios $\frac{a_{12}}{a_{11}}$ and $\frac{a_{21}}{a_{22}}$ on the outcome of the quantised consensus algorithm is also studied. Uniformly chosen initial conditions were used for the portion of the population which belonged to *X*, *Y* and *Z* respectively. Fig 5 depicts the percentage of the agents’ population that belonged to *X* with respect to the two ratios $\frac{a_{12}}{a_{11}}$ and $\frac{a_{21}}{a_{22}}$. The yellow corresponds to the cases where the whole population was in state *X* while the dark blue one corresponds to the cases where no agent was belonging to state *X*. As it can be observed, the highest the value of $\frac{a_{21}}{a_{22}}$ the highest the chances were to converge to *X* independently of the value of $\frac{a_{12}}{a_{11}}$.

The previous simulation results are for highly connected networks which can be considered as well mixed populations. In addition the structure of the network was randomly created. In order to study the behaviour of the proposed methodology in structured environments small world networks [41] were employed. On these ring-structured networks each agent is connected with $\frac{K}{2}$, 0<K≤N-1 nodes on each side of the ring. It has been showed [42–44], that the structure of the small world networks affects the speed of convergence of various consensus algorithms. Therefore it is possible because of the structure of small world networks that the quantised consensus algorithms and their corresponding Markov models will converge to different outcomes.

**Fig 6:**
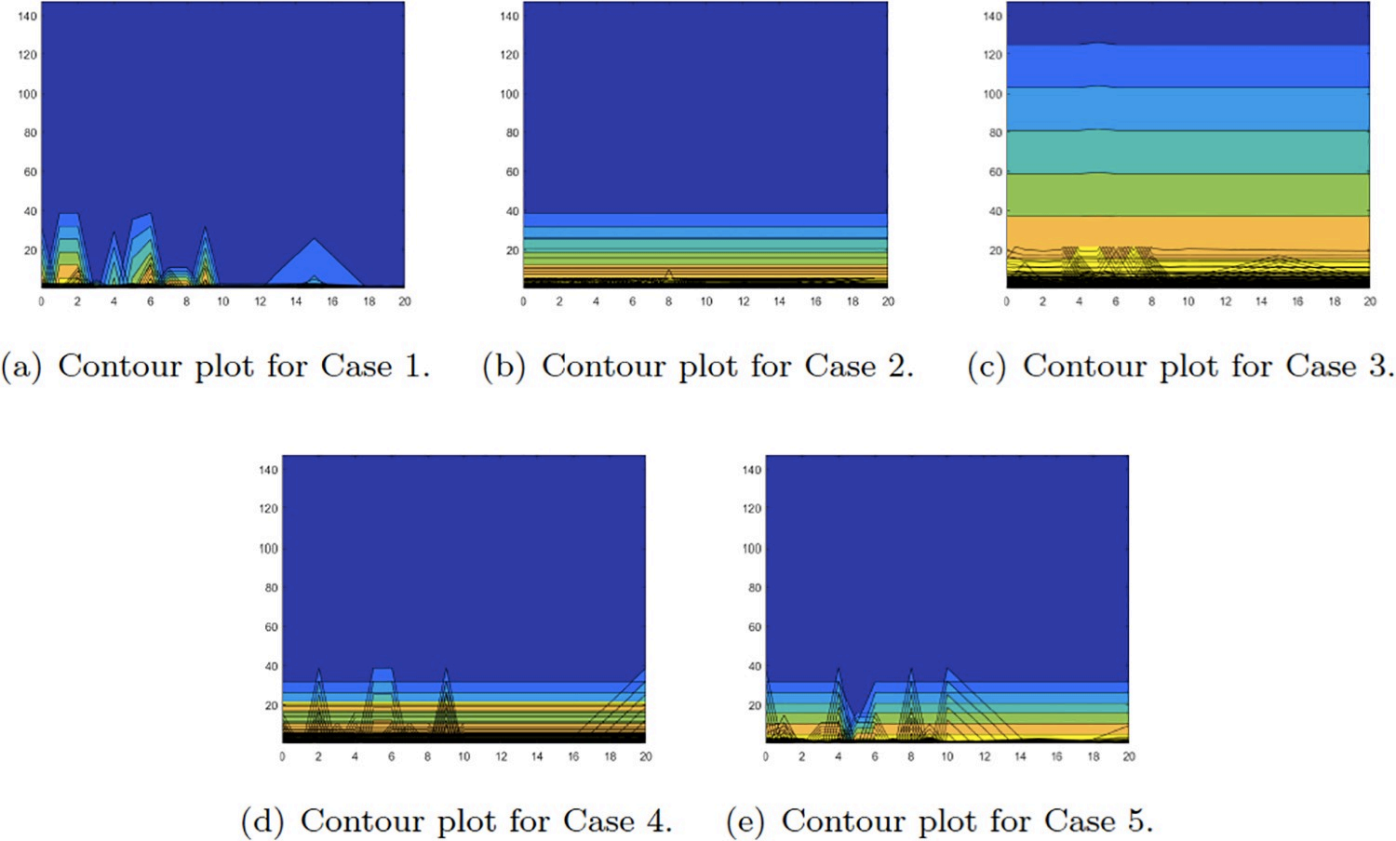
Simulation results for the *X* population when small world networks are considered. Dark blue colour denotes that quantised consensus and the markov chain resulted in the same decision in all the simulations. When yellow colour denotes that the results were the same only on the 40% of the cases. The *x*-axis corresponds to the minimum number of neighbours that an agent could have, in addition to the *m* neighbours which were necessary to have. The *y*-axis represents the various values of $\frac{a_{11}a_{21}}{a_{22}a_{12}}$.

In order to study the discrepancy between the quantised consensus and the Markov process in structured networks, the following experiments were employed. A Watts-Strogatz graph was created with 100 nodes connected with $\frac{K}{2}$ neighbours in each side and rewire probability *p*. The number of instances that both processes converged to the same decision were counted. These results are considered with regards to the number of connections that an agent can have and the impact that the reward function has. Since at least *m* neighbours are needed for the Cases 2-5, we have K=m+j,j∈{1,2,3,4,5,6,7,8,9,10,15,20}. In the simulations the case where *m* = 3 is considered. The simulations depicted in Fig 6 are of 100 replications of a network with the same K and random rewards *a*_*ij*_, *i*, *j* ∈ {1, 2}. In each replication both processes were repeated for 200 iterations. In order to take into account the importance of each opinion, and thus the rewards, the fraction $\frac{a_{11}a_{21}}{a_{22}a_{12}}$ is used. Which increases when opinion *X* is more important, i.e. has greater reward, and decreases when opinion *Y* is more important.

The results for case one are depicted in the first panel of Fig 6. The two processes result in the same decision as the value of $\frac{a_{11}a_{21}}{a_{22}a_{12}}$ increases and the number of neighbours K increases. The decisions of the two processed for the Cases 2,4 and 5, second fourth and fifth panel of Fig 6 respectively, depend more on the reward function rather than on K. On the other hand, in the third case the structured environment seems to influence the algorithms in a way that only when the importance of *X* is very high they converge to the same decision.

## 6 Discussion

The interconnection between agent-based decision-making and centralised decision-making through game theory is studied. In this article a scenario where agents that change their opinions depending on their peers is considered. Changes of opinions were modelled using a biology-inspired process based on the way bees choose the place of their next beehive.

Using the ideas of cross inhibitory signals, bees’ waggle dance and a quantised consensus process, different agent’s behaviours were formulated in the same model. This includes opinionated agents and crowd seeking or crowd averse agents. These behaviours were model based on the number of neighbours a person needed in order to change his opinion.

A game-theoretic approach was presented as a unifying formulation to the quantised consensus problem. Based on the expected gain pay-off function the quantised consensus process was cast as an evolutionary game. It was shown that for a game with three possible action “committed to opinion *X*”, “committed to opinion *Y*” and “not committed to any opinion” the game theoretic representation is equivalent to the quantised consensus process for well mixed populations.

The equilibria and their stability were analysed using the corresponding Markov process of each case. In all cases the whole population would eventually converge to a single opinion *X*, *Y* or *Z*. Exemption is the case of strong cross-inhibition signal where opinion *Z* is not a stable equilibrium. In this case it is also possible to observe a stable mixed equilibrium among the three opinions.

The impact that rewards have in the outcome of the processes have been also studied through simulations. In particular we have analysed the effect that $\frac{a_{12}}{a_{11}}$ and $\frac{a_{21}}{a_{22}}$ had in the decision process. In all cases there is an area where the process will always converge to a single opinion depending on which fraction is greater. Therefore if the rewards, value of an opinion, are sufficiently large the outcome will always converge to that opinion given a randomly chosen initial state of the population.

The validity of the results on structured networks was also studied through simulations, in small world networks. In the simulations of Case 1, if the rewards of an action and the number of an agent’s neighbours are sufficiently large, the quantised consensus process and the Markov process produce the same results. When Cases 2,4 and 5 are considered, similar results obtained when the reward of an action was excreting the other rewards by a certain level. The number of neighbour an agent had small or no effect on the number of instances that the two process produced the same results in those 3 cases. On the third case the number of neighbours is not mainly influences the results. Additionally, in this case the two processes have similar results only when there are big differences in the rewards of each action, i.e. $\frac{a_{11}a_{21}}{a_{22}a_{12}}>140$. These results indicate that for some of the cases studied it is possible to use the Markov process in structure environments as an alternative under specific conditions.

Among various future research directions an interesting extension of the current work is the study of an inhomogeneous case where some players are more important to their neighbours than others. In addition similarly to [45] the case where some “malicious” agents/players try to influence the equilibrium of the game will be studied.

## Supporting information

S1 File Quantised consensus models for each case. (PDF)

S2 File Proof of Theorem 1. (PDF)

S3 File Proof of Theorem 2. (PDF)

S4 File Corollary for the case *q* ≃ 1. (PDF)

S5 File Proof of Theorem 3. (PDF)

S6 File Appendix. (PDF)
