# Supplementary material for: An evolutionary game perspective on quantised consensus in opinion dynamics
Source: PLoS One. 2019 Jan 4;14(1):e0209212. doi: 10.1371/journal.pone.0209212 (PMC6319711; doi:10.1371/journal.pone.0209212)
Supplement: S1 File — (PDF) [file pone.0209212.s001.pdf]

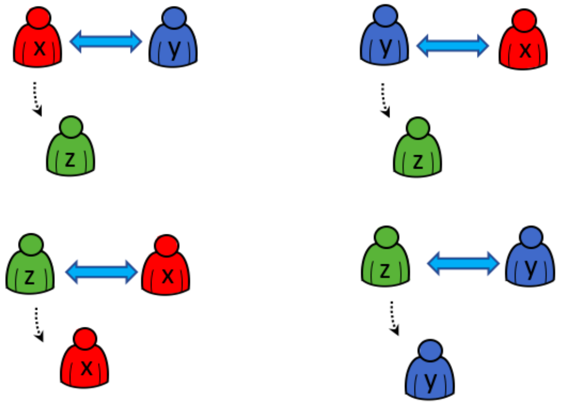

Fig 1. Possible mutations for Case 1.

S1 File S1 Quantised consensus models for each case.

Case 1: linear cross-inhibitory and waggle dance signal Consider the case where agents select randomly one of their neighbours and given to their current state, opinion, choose an action with certain probability. These can be considered as "broad minded" agents since they can reconsider their decisions by taking into account only the state of a single neighbour.

Fig. 1 is a graphical illustration of the opinion dynamics mechanism of Case 1. This case is characterised by linear waggle dance and cross-inhibitory signal. In the top-left, the reference agent is an individual who has opinion X (Individual X in short) and meets an individual with opinion Y (Individual Y in short) and changes his opinion into Z with probability $P_{XZ} = p_1$. When this occurs, one says that Individual X mutates into Individual Z. In the top-right, the reference agent is an individual with opinion Y who meets an individual with opinion X and updates his opinion into Z with probability $P_{YZ} = p_2$. In the bottom-left, the reference agent is now an individual with opinion Z who meets an individual with opinion X and changes his opinion into X with probability $P_{ZX} = p_3$. Finally, in the bottom-right, the reference agent is an individual with opinion Z who meets an individual with opinion Y and updates his opinion into Y with probability $P_{ZY} = p_4$.

The update mechanism of the reference agent i can be modelled as follows. Let w^i denote the state of the reference agent i. In addition, j will denote a randomly selected neighbour of agent i with state vale w^j. In the case where $w^i = X$ and $w^j = Y$, the reference agent changes his state value to $w^i = Z$ with probability p_1. Similarly, when $w^i = Y$, then i will change his value to $w^i = Z$ with probability p_2, if $w^j = X$. If $w^i = Z$, then i changes his value to $w^i = X$ with probability p_3 if $w^j = X$. The reference agent with state $w^i = Z$ mutates to $w^i = Y$ with probability p_4 if $w^j = Y$. Then the evolution of the decision variables is defined as follows:

$$w^i_{t+1}(w^i_t{=}X){=}\begin{cases} Z & \text{with probabilility } p_1, \text{ if } w^j_t{=}Y, \\ X & \text{otherwise;} \end{cases} \tag{1}$$

$$w^i_{t+1}(w^i_t{=}Y){=}\begin{cases} Z & \text{with probabilility } p_2, \text{ if } w^j_t{=}X, \\ Y & \text{otherwise;} \end{cases} \tag{2}$$

$$w^i_{t+1}(w^i_t{=}Z){=}\begin{cases} X & \text{with probabilility } p_3, \text{ if } w^j_t{=}X, \\ Y & \text{with probabilility } p_4, \text{ if } w^j_t{=}Y, \\ Z & \text{otherwise.} \end{cases} \tag{3}$$

The model (1)-(3) represents the microscopic model of Case 1, where the term microscopic indicates that the model refers to the reference agent i.

The microscopic model gives rise to a Markov process representation which captures the probability with which the reference agent mutates. Its generic form is given by

$$[x_{t+1} \quad y_{t+1} \quad z_{t+1}] = \underbrace{\begin{bmatrix} P_{XX} & P_{XY} & P_{XZ} \\ P_{YX} & P_{YY} & P_{YZ} \\ P_{ZX} & P_{ZY} & P_{ZZ} \end{bmatrix}}_{=:\mathbf{P}} [x_t \quad y_t \quad z_t]. \tag{4}$$

and the transition probability matrix is given by

$$\mathbf{P}_1 = \begin{bmatrix} (1-p_1)I_{jy} + (1-I_{jy}) & 0 & p_1 I_{jy} \\ 0 & (1-p_2)I_{jx} + (1-I_{jx}) & p_2 I_{jx} \\ p_3 I_{jx} & p_4 I_{jy} & (1-p_3)I_{jx} + (1-p_4)I_{jy} + I_{jz} \end{bmatrix}, \tag{5}$$

where I_{jx}, I_{jy}, and I_{jz} are the indicator functions establishing that neighbour j has value X, Y, Z, respectively, i.e.

$$I_{jx} = \begin{cases} 1 & \text{if } w_t^j = X, \\ 0 & \text{otherwise,} \end{cases} \quad I_{jy} = \begin{cases} 1 & \text{if } w_t^j = Y, \\ 0 & \text{otherwise,} \end{cases} \quad I_{jz} = \begin{cases} 1 & \text{if } w_t^j = Z, \\ 0 & \text{otherwise.} \end{cases} \tag{6}$$

Case 2: weak cross-inhibitory and strong waggle dance signal This second case deals with stubborn agents, who compare the decisions of more than one of their neighbours in order to change their opinion with a given probability. In terms of graph theory representation, it is assumed that each node is connected to at least m other nodes. The reference agent whose state value is either $w^i = X$ or $w^i = Y$ is willing to change his opinion only if m randomly chosen neighbours have a different opinion than his. Agents with decision variables $w^i = Z$ change opinions taking into account a single randomly selected neighbour.

All possible scenarios are illustrated in Fig. 2. In the top-left, the reference agent is an individual who has opinion X and meets with m neighbours with opinion Y and changes his opinion into Z with probability $P_{XZ} = p_1$. In the top-right, the reference agent has opinion Y and meets with m neighbours with opinion X. In consequence of this, he updates his opinion into Z with probability $P_{YZ} = p_2$. In the bottom-left, the reference agent is uncommitted, namely he has opinion Z, and after meeting an individual with opinion X updates his opinion into X with probability $P_{ZX} = p_3$. Finally, in the bottom-right, the reference agent has opinion Z; he meets with an individual with opinion Y and updates his opinion into Y with probability $P_{ZY} = p_4$.

To formulate the corresponding opinion dynamics mechanism, let $\mathcal{M}_t$ denote the set of i's neighbours with cardinality $|\mathcal{M}| \geq m$. Agent i randomly selects m neighbours at time t. Then the evolution of the decision variable for the aforementioned model is obtained as:

$$w_{t+1}^i(w_t^i{=}X){=}\begin{cases} Z & \text{with probabilility } p_1, \text{ if } w_t^j{=}Y, \forall j{\in}\mathcal{M}_t \\ X & \text{otherwise;} \end{cases} \tag{7}$$

$$w_{t+1}^i(w_t^i{=}Y){=}\begin{cases} Z & \text{with probabilility } p_2, \text{ if } w_t^j{=}X, \forall j{\in}\mathcal{M}_t, \\ Y & \text{otherwise;} \end{cases} \tag{8}$$

$$w_{t+1}^i(w_t^i{=}Z){=}\begin{cases} X & \text{with probabilility } p_3, \text{ if } w_t^j{=}X, \\ Y & \text{with probabilility } p_4, \text{ if } w_t^j{=}Y, \\ Z & \text{otherwise.} \end{cases} \tag{9}$$

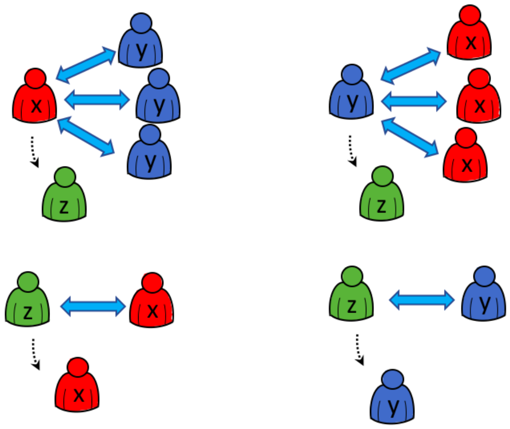

Fig 2. Possible mutations for Case 2.

The microscopic model (7)-(9) gives rise to a Markov process representation as in (4), which captures the probability with which the reference agent mutates. The transition probability matrix is given by

$$\mathbf{P}_2 = \begin{bmatrix} (1-p_1)I_{My} + (1-I_{My}) & 0 & p_1 I_{My} \\ 0 & (1-p_2)I_{Mx} + (1-I_{Mx}) & p_2 I_{Mx} \\ p_3 I_{jx} & p_4 I_{jy} & (1-p_3)I_{jx} + (1-p_4)I_{jy} + I_{jz} \end{bmatrix}. \quad (10)$$

In the above, I_{jx}, I_{jy} and I_{jz} are as in (6), and we also denote I_{Mx} and I_{My} as the indicator functions describing the cases where all the neighbours are in state X or state Y, respectively. More formally,

$$I_{Mx} = \begin{cases} 1 & \text{if } w_t^j = X, \quad \forall j \in \mathcal{M}_t, \\ 0 & \text{otherwise,} \end{cases} \qquad I_{My} = \begin{cases} 1 & \text{if } w_t^j = Y, \quad \forall j \in \mathcal{M}_t, \\ 0 & \text{otherwise.} \end{cases} \quad (11)$$

Case 3: strong cross-inhibitory and weak waggle dance signal The third case deals with stubbornness of uncommitted agents. Consider a reference agent who is not committed, i.e. his opinion is $w^i = Z$. He commits to opinion X or Y only if m randomly chosen neighbours have opinion X or Y, respectively. Committed agents use the opinion of a single randomly chosen neighbour.

This is illustrated in Fig. 3. In the top-left, the reference agent has opinion X and after meeting with a random neighbour with opinion Y, he changes his opinion into Z with probability $P_{XZ} = p_1$. In the top-right, a reference agent with opinion Y randomly selects a neighbour and who has opinion X. Then he updates his opinion into Z with probability $P_{YZ} = p_2$. In the bottom-left, the reference agent is uncommitted (opinion Z) and because all m neighbours have opinion X, he changes his opinion into X with probability $P_{ZX} = p_3$. In the bottom-right, the reference agent has again opinion Z but now he meets m neighbours with opinion Y. He then updates his opinion into Y with probability $P_{ZY} = p_4$.

The evolution of the opinions for this case is described by the following microscopic model:

$$w_{t+1}^i(w_t^i{=}X){=}\begin{cases} Z & \text{with probabilility } p_1, \text{ if } w_t^j{=}Y, \\ X & \text{otherwise;} \end{cases} \quad (12)$$

$$w_{t+1}^i(w_t^i{=}Y){=}\begin{cases} Z & \text{with probabilility } p_2, \text{ if } w_t^j{=}X, \\ Y & \text{otherwise;} \end{cases} \quad (13)$$

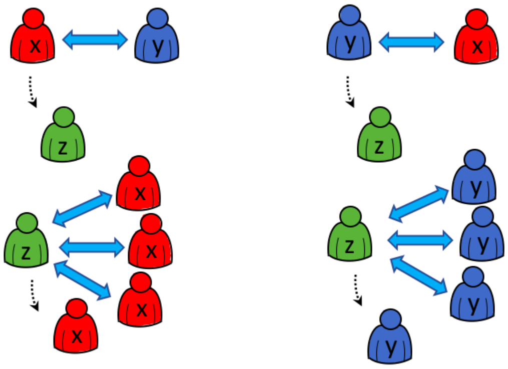

Fig 3. Possible mutations for Case 3.

$$w^i_{t+1}(w^i_t{=}Z){=}\begin{cases} X & \text{with probabilility } p_3, \text{ if } w^j_t{=}X, \forall j{\in}\mathcal{M}_t, \\ Y & \text{with probabilility } p_4, \text{ if } w^j_t{=}Y, \forall j{\in}\mathcal{M}_t, \\ Z & \text{otherwise.} \end{cases} \tag{14}$$

The above microscopic model (12)-(14) yields a Markov process representation as in (4). The transition probability matrix is given by

$$\mathbf{P}_3 = \begin{bmatrix} (1-p_1)I_{jy} + (1-I_{jy}) & 0 & p_1 I_{jy} \\ 0 & (1-p_2)I_{jx} + (1-I_{jx}) & p_2 I_{jx} \\ p_3 I_{Mx} & p_4 I_{My} & (1-p_3)I_{Mx} + (1-p_4)I_{My} + \tilde{I}_{Mxy} \end{bmatrix}. \tag{15}$$

In the above we use the notation I_{jx} and I_{jy} from (6) and I_{Mx} and I_{My} from (11), while $\tilde{I}_{Mxy}$ denotes the indicator function which establishes that both $I_{Mx} = 0$ and $I_{My} = 0$. This can be formulated as $\tilde{I}_{Mxy} \in \{0, 1\}$ such that $\tilde{I}_{Mxy} = 1 - I_{Mx} - I_{My}$.

Case 4: neighbor-based cross-inhibitory signal The fourth case involves agents who make decisions by taking into account the percentage of their neighbours which belong to X and Y only. Let $\tilde{x}^i$ and $\tilde{y}^i$ denote the percentage of agent i's neighbours whose decision variable is X and Y respectively. The probability of changing opinion is divided by $\frac{1}{\tilde{x}^i+\tilde{y}^i}$. Only transitions to the uncommitted state Z, are evaluated using this process as it is illustrated in Fig. 4.

In the top-left, the reference agent has opinion X and his random neighbour has opinion Y. He then changes his opinion into Z with probability $P_{XZ} = p_1 \frac{1}{\tilde{x}^i+\tilde{y}^i}$. In the top-right, a reference agent with opinion Y randomly selects a neighbour and this has opinion X. Then he changes opinion into Z with probability $P_{YZ} = p_2 \frac{1}{\tilde{x}^i+\tilde{y}^i}$. In the bottom-left, the reference agent is uncommitted (opinion Z) and because his neighbour has opinion X, he changes his opinion into X with probability $P_{ZX} = p_3$. In the bottom-right, the reference agent has opinion Z but now he meets a neighbour with opinion Y and updates his opinion into Y with probability $P_{ZY} = p_4$.

The microscopic model which captures the evolution of w is obtained as follows:

$$w^i_{t+1}(w^i_t{=}X){=}\begin{cases} Z & \text{with prob. } p_1 \frac{1}{\tilde{x}^i+\tilde{y}^i}, \text{ if } w^j_t{=}Y, \\ X & \text{otherwise;} \end{cases} \tag{16}$$

$$w^i_{t+1}(w^i_t{=}X){=}\begin{cases} Z & \text{with prob. } p_2 \frac{1}{\tilde{x}^i+\tilde{y}^i}, \text{ if } w^j_t{=}X, \\ Y & \text{otherwise;} \end{cases} \tag{17}$$

$$w^i_{t+1}(w^i_t{=}Z){=}\begin{cases} X & \text{with probabilility } p_3, \text{ if } w^j_t{=}X, \\ Y & \text{with probabilility } p_4, \text{ if } w^j_t{=}Y, \\ Z & \text{otherwise.} \end{cases} \tag{18}$$

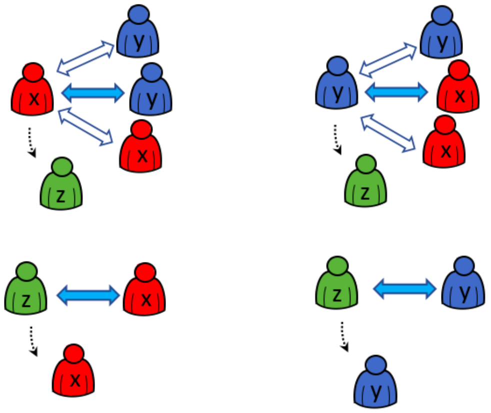

Fig 4. Possible mutations for Case 4.

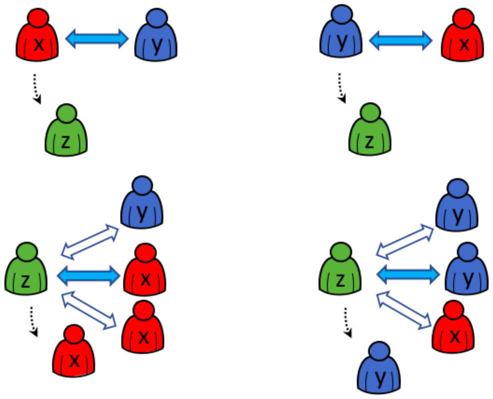

Fig 5. Possible mutations for Case 5.

The above microscopic model (16)-(18) yields a Markov process representation as in (4). The transition probability matrix is given by

$$\mathbf{P}_4 = \begin{bmatrix} (1 - p_1 \frac{1}{\tilde{x}^i + \tilde{y}^i}) I_{jy} + (1 - I_{jy}) & 0 & p_1 \frac{1}{\tilde{x}^i + \tilde{y}^i} I_{jy} \\ 0 & (1 - p_2 \frac{1}{\tilde{x}^i + \tilde{y}^i}) I_{jx} + (1 - I_{jx}) & p_2 \frac{1}{\tilde{x}^i + \tilde{y}^i} I_{jx} \\ p_3 I_{jx} & p_4 I_{jy} & (1 - p_3) I_{jx} + (1 - p_4) I_{jy} + I_{jz} \end{bmatrix}. \quad (19)$$

The notation for I_{jx} and I_{jy} and I_{jz} is as in (6).

Case 5: neighbor-based waggle dance signal The last case deals with stubborn uncommitted players who commit to an opinion based on the number of neighbours committed to X and Y. This is depicted in Fig. 5. In the top-left, a reference agent with opinion X meets a random neighbour with opinion Y and changes his opinion into Z with probability $P_{XZ} = p_1$. In the top-right, a reference agent with opinion Y meets a random neighbour with opinion X and changes opinion into Z with probability $P_{YZ} = p_2$. In the bottom-left, the reference agent is uncommitted (opinion Z), he meets a neighbour with opinion X, and changes his opinion into X with probability $P_{ZX} = p_3 \frac{1}{\tilde{x}^i + \tilde{y}^i}$, which depends on the numbers of individuals X and Y in his neighbourhood. In the bottom-right, the reference agent has opinion Z, he meets a neighbour with opinion Y and updates his opinion into Y with probability $P_{ZY} = p_4 \frac{1}{\tilde{x}^i + \tilde{y}^i}$, which depends on the numbers of individuals X and Y in his neighbourhood.

The microscopic model which describes the opinion dynamics of the reference agent is

$$w_{t+1}^i(w_t^i{=}X){=}\begin{cases} Z & \text{with probabilility } p_1, \text{ if } w_t^j{=}Y, \\ X & \text{otherwise;} \end{cases} \tag{20}$$

$$w_{t+1}^i(w_t^i{=}Y){=}\begin{cases} Z & \text{with probabilility } p_2, \text{ if } w_t^j{=}X, \\ Y & \text{otherwise;} \end{cases} \tag{21}$$

$$w_{t+1}^i(w_t^i{=}Z){=}\begin{cases} X & \text{with probabilility } p_3\frac{1}{\tilde{x}^i+\tilde{y}^i}, \text{ if } w_t^j{=}X, \\ Y & \text{with probabilility } p_4\frac{1}{\tilde{x}^i+\tilde{y}^i}, \text{ if } w_t^j{=}Y, \\ Z & \text{otherwise.} \end{cases} \tag{22}$$

This results a Markov process representation as in (4), which describes the probability distribution over the opinions. The transition probability matrix the is given by:

$$\mathbf{P}_5 = \begin{bmatrix} (1-p_1)I_{jy} + (1-I_{jy}) & 0 & p_1 I_{jy} \\ 0 & (1-p_2)I_{jx} + (1-I_{jx}) & p_2 I_{jx} \\ p_3\frac{1}{\tilde{x}^i+\tilde{y}^i}I_{jx} & p_4\frac{1}{\tilde{x}^i+\tilde{y}^i}I_{jy} & (1-p_3\frac{1}{\tilde{x}^i+\tilde{y}^i})I_{jx} + (1-p_4\frac{1}{\tilde{x}^i+\tilde{y}^i})I_{jy} + I_{jz} \end{bmatrix}, \tag{23}$$

where the I_{jx} and I_{jy} and I_{jz} are defined as in (6).
