# Supplementary material for: An evolutionary game perspective on quantised consensus in opinion dynamics
Source: PLoS One. 2019 Jan 4;14(1):e0209212. doi: 10.1371/journal.pone.0209212 (PMC6319711; doi:10.1371/journal.pone.0209212)
Supplement: S2 File — (PDF) [file pone.0209212.s002.pdf]

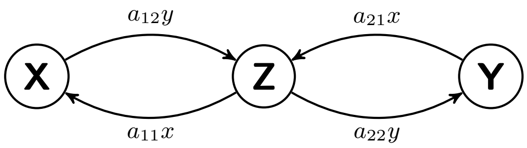

Figure 1: Markov chain with transition probabilities linearly depending on x and y (Case 1).

S2 File Proof of Theorem 1

Proof. We prove the above result for each of the five cases.
(Case 1) The transition probabilities from and to Z are linear functions of x and y respectively, with reward functions defined as:

$$\text{(Case 1)} \qquad A = \begin{array}{c} \\ X \\ Y \\ Z \end{array} \begin{array}{c} \begin{array}{ccc} X & Y & Z \end{array} \\ \begin{pmatrix} a_{11} & -a_{12} & 0 \\ -a_{21} & a_{22} & 0 \\ 0 & 0 & 0 \end{pmatrix} \end{array}. \tag{1}$$

Then the probabilities with which the players decide to change action, based on the expected gain pairwise comparison

$$E_{ij} = \sum_{k=1}^{k=n} \mathcal{I}(a_{ik} - a_{jk})x_k, \tag{2}$$

are given by

$$\begin{array}{ll} p_{ZX} = a_{11}x, & p_{XZ} = a_{12}y, \\ p_{YZ} = a_{21}x, & p_{ZY} = a_{22}y. \end{array} \tag{3}$$

Note here that these transition rates will be probabilities as far as $0 \leq p_{XZ}, p_{ZX}, p_{YZ}, p_{ZN} \leq 1$. This is guaranteed if $0 \leq a_{11}, a_{12}, a_{21}, a_{22} \leq 1$. The evolution of x, y and z can be described by the Markov process depicted in Fig. 1. The evolution of x, y and z according to the aforementioned Markov process is:

$$\begin{array}{rl} x_{t+1} = & x_t - a_{12}y_t x_t + a_{11}x_t(1 - x_t - y_t), \\ y_{t+1} = & y_t - a_{21}y_t x_t + a_{22}y_t(1 - x_t - y_t). \end{array} \tag{4}$$

It is left to prove that (4) is the macroscopic dynamics obtained from the following microscopic consensus dynamics:

$$w_{t+1}^i(w_t^i{=}X){=}\begin{cases} Z & \text{with probabilility } p_1, \text{ if } w_t^j{=}Y, \\ X & \text{otherwise;} \end{cases} \tag{5}$$

$$w_{t+1}^i(w_t^i{=}Y){=}\begin{cases} z & \text{with probabilility } p_2, \text{ if } w_t^j{=}X, \\ Y & \text{otherwise;} \end{cases} \tag{6}$$

$$w_{t+1}^i(w_t^i{=}Z){=}\begin{cases} X & \text{with probabilility } p_3, \text{ if } w_t^j{=}X, \\ Y & \text{with probabilility } p_4, \text{ if } w_t^j{=}Y, \\ Z & \text{otherwise.} \end{cases} \tag{7}$$

To this purpose, let $W_t(x), W_t(y)$ and $W_t(z)$ be the portion of agents who are in X, Y and Z at time t, respectively. Note that $W_t(x) + W_t(y) + W_t(z) = 1$. In a well mixed population the probability that one of the neighbours of an agent i has opinion either X, Y or Z is $W_t(x), W_t(y)$ and

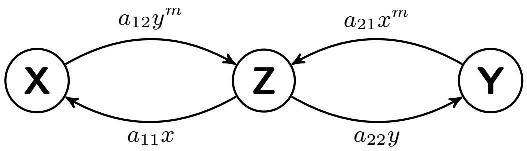

Figure 2: Markov chain emerging from the case of a weak cross-inhibitory signal (Case 2).

$W_t(z)$, respectively. Then, under the generic probabilities p_1, p_2, p_3, p_4 the evolution of the three populations are:

$$
\begin{aligned}
W_{t+1}(x) &= W_t(x) - p_1 W_t(x) W_t(y) + p_3 W_z, \\
W_{t+1}(y) &= W_t(y) - p_2 W_t(y) W_t(x) + p_4 W_z, \\
W_{t+1}(z) &= W_t(z) - p_3 W_t(z) W_t(x) - p_4 W_z(t) W_t(y) + p_1 W_t(x) W_t(y) + p_2 W_t(x) W_t(y).
\end{aligned}
\tag{8}
$$

If the probabilities p_1, p_2, p_3 and p_4 are set equal to $a_{12}W_t(y), a_{21}W_t(x), a_{11}W_t(x)$ and $a_{22}W_t(y)$, the evolution equations (4) and (8) are identical which concludes the proof for Case 1.

(Case 2) The probability in a well mixed population to randomly meet m individuals whose actions are X or Y are $(W_t(x))^m$ and $(W_t(y))^m$ respectively. Therefore, the cross-inhibitory signals, $f_2(y)$ and $f_4(x)$ are the mth power of y and x respectively, and the transitions from z will be linear functions with respect to x and y respectively. Since $x, y \leq 1$ the transition probabilities towards the neutral opinion will be smaller if compared with the ones that are linear functions of y and x. The reward function for the weak cross-inhibitory signal case is then defined as:

$$
\text{(Case 2)} \qquad A(x,y) = \begin{array}{c} \\ X \\ Y \\ Z \end{array} \begin{array}{c} \begin{array}{ccc} X & Y & Z \end{array} \\ \begin{pmatrix} a_{11} & -a_{12}y^{m-1} & 0 \\ -a_{21}x^{m-1} & a_{22} & 0 \\ 0 & 0 & 0 \end{pmatrix} \end{array}. \tag{9}
$$

The evolution of the population of X, Y and Z according to (15) is:

$$
\begin{aligned}
x_{t+1} = &\quad x_t - a_{12} y_t^m x_t + a_{11} x_t (1 - x_t - y_t), \\
y_{t+1} = &\quad y_t - a_{21} y_t x_t^m + a_{22} y_t (1 - x_t - y_t),
\end{aligned}
\tag{10}
$$

and it is depicted in Fig. 2.

Let us now show that the macroscopic description (10) is consistent with the following microscopic consensus dynamics:

$$
w_{t+1}^i(w_t^i{=}X){=}\begin{cases} Z & \text{with probabilility } p_1, \text{ if } w_t^j{=}Y, \forall j{\in}\mathcal{M}_t \\ X & \text{otherwise;} \end{cases} \tag{11}
$$

$$
w_{t+1}^i(w_t^i{=}Y){=}\begin{cases} Z & \text{with probabilility } p_2, \text{ if } w_t^j{=}X, \forall j{\in}\mathcal{M}_t, \\ Y & \text{otherwise;} \end{cases} \tag{12}
$$

$$
w_{t+1}^i(w_t^i{=}Z){=}\begin{cases} X & \text{with probabilility } p_3, \text{ if } w_t^j{=}X, \\ Y & \text{with probabilility } p_4, \text{ if } w_t^j{=}Y, \\ Z & \text{otherwise.} \end{cases} \tag{13}
$$

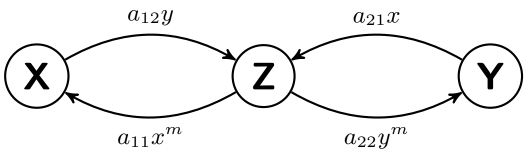

Figure 3: Markov chain emerging from the case of a strong cross-inhibitory signal (Case 3).

Given the probabilities p_1, p_2, p_3, p_4 the evolution of the populations are:

$$\begin{aligned} W_{t+1}(x) &= W_t(x) - p_1 W_t(x)(W_t(y))^m + p_3 W_z, \\ W_{t+1}(y) &= W_t(y) - p_2 W_t(y)(W_t(x))^m + p_4 W_z, \\ W_{t+1}(z) &= W_t(z) - p_3 W_t(z)(W_t(x) - p_4 W_z(t)(W_t(y) \\ &\quad + p_1 W_t(x)(W_t(y))^m + p_2 (W_t(x))^m W_t(y). \end{aligned} \tag{14}$$

For a well mixed population, and since the probability that one neighbour belongs to one category is independent of other neighbours belonging to the same category, the transition probabilities are defined as $p_1 = a_{12}(\frac{W_y}{\mathcal{N}})^m$. The probabilities p_2, p_3 and p_4 are defined as: $p_2 = a_{21}(\frac{W_x}{\mathcal{N}})^m$, $p_3 = a_{11}\frac{W_z}{\mathcal{N}}$ and $p_4 = a_{22}\frac{W_z}{\mathcal{N}}$. The evolution equations (10) and (14) are identical which concludes the proof for Case 2.

(Case 3) The transition rates from Z are probability distribution powers of x and y, namely $x^m, y^m, m \geq 2$. The reward function for the strong cross-inhibitory signal case is then defined as:

$$\text{(Case 3)} \qquad A(x,y) = \begin{matrix} \\ X \\ Y \\ Z \end{matrix} \begin{matrix} \begin{matrix} X & \qquad Y & \qquad Z \end{matrix} \\ \begin{pmatrix} a_{11}x^{m-1} & -a_{12} & 0 \\ -a_{21} & a_{22}y^{m-1} & 0 \\ 0 & 0 & 0 \end{pmatrix} \end{matrix}, \tag{15}$$

and the corresponding Markov process is depicted in Fig. 3.

The evolution of the population of X, Y and Z according to the aforementioned Markov process is:

$$\begin{aligned} x_{t+1} &= \quad x_t - a_{12}y_t x_t + a_{11}x_t^m(1 - x_t - y_t), \\ y_{t+1} &= \quad y_t - a_{21}y_t x_t + a_{22}y_t^m(1 - x_t - y_t). \end{aligned} \tag{16}$$

We have to prove now that the population behaviour (16) is consistent with the following consensus dynamics for the reference player:

$$w_{t+1}^i(w_t^i{=}X){=}\begin{cases} Z & \text{with probabilility } p_1, \text{ if } w_t^j{=}Y, \\ X & \text{otherwise;} \end{cases} \tag{17}$$

$$w_{t+1}^i(w_t^i{=}Y){=}\begin{cases} Z & \text{with probabilility } p_2, \text{ if } w_t^j{=}X, \\ Y & \text{otherwise;} \end{cases} \tag{18}$$

$$w_{t+1}^i(w_t^i{=}Z){=}\begin{cases} X & \text{with probabilility } p_3, \text{ if } w_t^j{=}X, \forall j{\in}\mathcal{M}_t, \\ Y & \text{with probabilility } p_4, \text{ if } w_t^j{=}Y, \forall j{\in}\mathcal{M}_t, \\ Z & \text{otherwise.} \end{cases} \tag{19}$$

Similarly to the strong cross-inhibitory signal case, given the probabilities p_1, p_2, p_3, p_4 the evolution of the three populations are:

$$\begin{aligned} W_{t+1}(x) &= W_t(x) - p_1 W_t(x)(W_t(y))^m + p_3 W_z \\ W_{t+1}(y) &= W_t(y) - p_2 W_t(y)(W_t(x))^m + p_4 W_z \\ W_{t+1}(z) &= W_t(z) - p_3 W_t(z)(W_t(x) - p_4 W_z(t)(W_t(y) \\ &\quad + p_1 W_t(x)(W_t(y))^m + p_2 (W_t(x))^m W_t(y), \end{aligned} \tag{20}$$

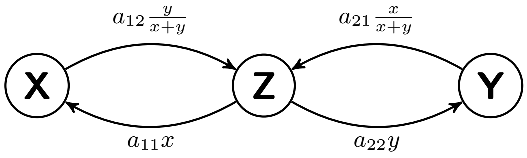

Figure 4: Model with transition probabilities from X and Y to Z depending on x and y respectively.

where the probabilities p_1 p_2, p_3 and p_4 are defined as: $p_1 = a_{12}\frac{W_y}{\mathcal{N}}$, $p_2 = a_{21}\frac{W_x}{\mathcal{N}}$, $p_3 = a_{11}(\frac{W_x}{\mathcal{N}})^m\frac{W_z}{\mathcal{N}}$ and $p_4 = a_{22}(\frac{W_y}{\mathcal{N}})^m\frac{W_z}{\mathcal{N}}$. The evolution equations (16) and (20) are identical which concludes the proof for Case 3.

(Case 4) In this case the transition probabilities towards Z, are higher than the transition probabilities back from Z to X and Y. These probabilities are defined as fractions of players choosing X and players choosing Y in a fictitious population where only cooperators and defectors exist. This results in the rewards functions which are given by:

$$\text{(Case 4)} \qquad A(x,y) = \begin{array}{c} \\ X \\ Y \\ Z \end{array} \begin{array}{c} \begin{array}{ccc} X & Y & Z \end{array} \\ \begin{pmatrix} a_{11} & -a_{12}\frac{1}{x+y} & 0 \\ -a_{21}\frac{1}{x+y} & a_{22} & 0 \\ 0 & 0 & 0 \end{pmatrix} \end{array}. \tag{21}$$

The corresponding Markov process is depicted in Fig. 4.

The evolution of the population of X, Y is then obtained as:

$$\begin{aligned} x_{t+1} &= \quad x_t - a_{12}\frac{y_t x_t}{x_t+y_t} + a_{11}x_t(1-x_t-y_t), \\ y_{t+1} &= \quad y_t - a_{21}\frac{y_t x_t}{x_t+y_t} + a_{22}y_t(1-x_t-y_t). \end{aligned} \tag{22}$$

We are now left with proving that (22) is consistent with the foolowing consensus dynamics:

$$w^i_{t+1}(w^i_t{=}X){=}\begin{cases} Z & \text{with prob. } p_1\frac{1}{\tilde{x}^i+\tilde{y}^i}\text{, if } w^j_t{=}Y, \\ X & \text{otherwise;} \end{cases} \tag{23}$$

$$w^i_{t+1}(w^i_t{=}X){=}\begin{cases} Z & \text{with prob. } p_2\frac{1}{\tilde{x}^i+\tilde{y}^i}\text{, if } w^j_t{=}X, \\ Y & \text{otherwise;} \end{cases} \tag{24}$$

$$w^i_{t+1}(w^i_t{=}Z){=}\begin{cases} X & \text{with probabilility } p_3\text{, if } w^j_t{=}X, \\ Y & \text{with probabilility } p_4\text{, if } w^j_t{=}Y, \\ Z & \text{otherwise.} \end{cases} \tag{25}$$

Given the probabilities p_1, p_2, p_3, p_4 the evolution of the three populations are:

$$\begin{aligned} W_{t+1}(x) \quad &= W_t(x) - p_1 W_t(x)W_t(y)\frac{1}{\tilde{x}+\tilde{y}} + p_3 W_z \\ W_{t+1}(y) \quad &= W_t(y) - p_2 W_t(y)W_t(x)\frac{1}{\tilde{x}+\tilde{y}} + p_4 W_z \\ W_{t+1}(z) \quad &= W_t(z) - p_3 W_t(z)W_t(x) - p_4 W_z(t)W_t(y) \\ &\quad + p_1 W_t(x)W_t(y)\frac{1}{\tilde{x}+\tilde{y}} + p_2 W_t(x)\frac{1}{\tilde{x}+\tilde{y}}W_t(y). \end{aligned} \tag{26}$$

For a well mixed population, the probabilities p_1 p_2, p_3 and p_4 are defined as: $p_1 = a_{12}\frac{\mathcal{N}}{W_x+W_y}\frac{W_y}{\mathcal{N}}$, $p_2 = a_{21}\frac{\mathcal{N}}{W_x+W_y}\frac{W_x}{\mathcal{N}}$, $p_3 = a_{11}\frac{W_z}{\mathcal{N}}$ and $p_4 = a_{22}\frac{W_z}{\mathcal{N}}$, which concludes the proof for Case 4.

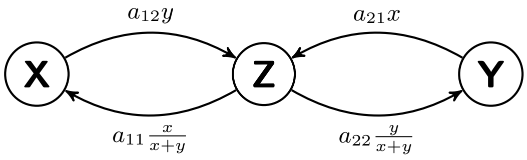

Figure 5: Model with transition probabilities from Z to X and Y depending on x and y.

(Case 5) In this case the transition probabilities from Z, are higher than the transition probabilities towards Z from X and Y. These probabilities are defined as fractions of players choosing X and players choosing Y in a fictitious population where only cooperators and defectors exist. This results in the rewards functions which are given by:

$$\text{(Case 5)} \qquad A(x,y) = \begin{array}{c} \\ X \\ Y \\ Z \end{array} \begin{array}{c} \begin{array}{ccc} X & Y & Z \end{array} \\ \begin{pmatrix} a_{11}\frac{1}{x+y} & -a_{12} & 0 \\ -a_{21} & a_{22}\frac{1}{x+y} & 0 \\ 0 & 0 & 0 \end{pmatrix} \end{array}. \tag{27}$$

The corresponding Markov process is depicted in Fig. 5.

The evolution of the populations X, Y and Z according to the aforementioned Markov process is:

$$\begin{aligned} x_{t+1} = &\quad x - a_{12}y_t x_t + a_{11}\tfrac{x_t}{x_t+y_t}(1 - x_t - y_t), \\ y_{t+1} = &\quad y - a_{21}y_t x_t + a_{22}\tfrac{y_t}{x_t+y_t}(1 - x_t - y_t). \end{aligned} \tag{28}$$

Let us now prove that the set of equations in (28) are consistent with the following:

$$w^i_{t+1}(w^i_t{=}X){=}\begin{cases} Z & \text{with probabilility } p_1, \text{ if } w^j_t{=}Y, \\ X & \text{otherwise;} \end{cases} \tag{29}$$

$$w^i_{t+1}(w^i_t{=}Y){=}\begin{cases} Z & \text{with probabilility } p_2, \text{ if } w^j_t{=}X, \\ Y & \text{otherwise;} \end{cases} \tag{30}$$

$$w^i_{t+1}(w^i_t{=}Z){=}\begin{cases} X & \text{with probabilility } p_3\frac{1}{\tilde{x}^i+\tilde{y}^i}, \text{ if } w^j_t{=}X, \\ Y & \text{with probabilility } p_4\frac{1}{\tilde{x}^i+\tilde{y}^i}, \text{ if } w^j_t{=}Y, \\ Z & \text{otherwise.} \end{cases} \tag{31}$$

Given the probabilities p_1, p_2, p_3, p_4 the evolution of the three populations are:

$$\begin{aligned} W_{t+1}(x) &= W_t(x) - p_1 W_t(x)W_t(y) + p_3 W_z\tfrac{1}{\tilde{x}+\tilde{y}} \\ W_{t+1}(y) &= W_t(y) - p_2 W_t(y)W_t(x) + p_4 W_z\tfrac{1}{\tilde{x}+\tilde{y}} \\ W_{t+1}(z) &= W_t(z) - p_3 W_t(z)W_t(x)\tfrac{1}{\tilde{x}+\tilde{y}} \\ &\quad -p_4 W_z(t)W_t(y)\tfrac{1}{\tilde{x}+\tilde{y}} + p_1 W_t(x)W_t(y) + p_2 W_t(x)W_t(y). \end{aligned} \tag{32}$$

For a well mixed population the probabilities p_1 p_2, p_3 and p_4 are defined as: $p_1 = a_{12}\frac{W_y}{\mathcal{N}}$, $p_2 = a_{21}\frac{W_x}{\mathcal{N}}$, $p_3 = a_{11}\frac{\mathcal{N}}{W_x+W_y}\frac{W_z}{\mathcal{N}}$ and $p_4 = a_{22}\frac{\mathcal{N}}{W_x+W_y}\frac{W_z}{\mathcal{N}}$. This concludes our proof. □
