# Supplementary material for: An evolutionary game perspective on quantised consensus in opinion dynamics
Source: PLoS One. 2019 Jan 4;14(1):e0209212. doi: 10.1371/journal.pone.0209212 (PMC6319711; doi:10.1371/journal.pone.0209212)
Supplement: S3 File — (PDF) [file pone.0209212.s003.pdf]

S3 file   Proof of theorem 2

Theorem. *The following tuples are equilibrium points for the evolutionary game (8A unified framework based on evolutionary game-theoretic formulationequation.3.8):*

$$(x=1, y=0, z=0), \quad (x=0, y=1, z=0), \quad (x=0, y=0, z=1).$$

In addition, a fourth equilibrium point may exist of type $(x, qx, 1-(1+q)x)$. *In particular,*

- *Cases 1, 4, and 5: it holds* $q = \frac{a_{11}a_{21}}{a_{12}a_{22}}$ *and the equilibrium* $(x, qx, 1-(1+q)x)$ *is given by*

$$\text{(Case 1)}\Big(x = \frac{a_{11}}{a_{12}q + a_{11}(1+q)},\ y = q\frac{a_{11}}{a_{12}q + a_{11}(1+q)}, \tag{1}$$

$$z = 1 - (1+q)\frac{a_{11}}{a_{12}q + a_{11}(1+q)}\Big), \tag{2}$$

$$\text{(Case 4)}\Big(x = \frac{a_{11}(1+q) - a_{12}q}{a_{11}(1+q^2)},\ y = q\frac{a_{11}(1+q) - a_{12}q}{a_{11}(1+q^2)}, \tag{3}$$

$$z = 1 - (1+q)\frac{a_{11}(1+q) - a_{12}q}{a_{11}(1+q^2)}\Big), \tag{4}$$

$$\text{(Case 5)}\Big(x = \frac{-a_{11}(1+q) + \sqrt{a_{11}^2(1+q)^2 + 4a_{12}(q^2+q)a_{11}}}{2a_{12}(q^2+q)},\ y = q\frac{-a_{11}(1+q) + \sqrt{a_{11}^2(1+q)^2 + 4a_{12}(q^2+q)a_{11}}}{2a_{12}(q^2+q)},$$

$$z = 1 - (1+q)\frac{a_{11}(1+q) - a_{12}q}{a_{11}(1+q^2)}\Big). \tag{5}$$

- *Cases 2 and 3: it holds* $q = \sqrt[m]{\frac{a_{11}a_{21}}{a_{12}a_{22}}}$ *and the equilibrium* $(x, qx, 1-(1+q)x)$ *is given by*

$$\text{(Case 2)}\Big(x = \frac{-a_{11}(1+q) + \sqrt{a_{11}^2(1+q)^2 + 4a_{12}q^2a_{11}}}{2a_{12}q^2}, \tag{6}$$

$$y = q\frac{-a_{11}(1+q) + \sqrt{a_{11}^2(1+q)^2 + 4a_{12}q^2a_{11}}}{2a_{12}q^2},$$

$$z = 1 - (1+q)\frac{-a_{11}(1+q) + \sqrt{a_{11}^2(1+q)^2 + 4a_{12}q^2a_{11}}}{2a_{12}q^2}\Big),\ m = 2,$$

$$\Big(x = \frac{a_{11} - \epsilon_m}{a_{11}(1+q)},\ y = q\frac{a_{11} - \epsilon_m}{a_{11}(1+q)},\ z = 1 - \frac{a_{11} - \epsilon_m}{a_{11}}\Big),\ \lim_{m\to\infty}\epsilon_m = 0,\ m \text{ large}, \tag{7}$$

$$\text{(Case 3)}\Big(x = \frac{a_{11} - a_{12}q}{a_{11}(1+q)},\ y = q\frac{a_{11} - a_{12}q}{a_{11}(1+q)},\ z = 1 - (1+q)\frac{a_{11} - a_{12}q}{a_{11}(1+q)}\Big), m = 2, \tag{8}$$

$$\Big(x = \frac{a_{11} \pm \sqrt{a_{11}^2(1+q)^2 - 4a_{11}(1+q)a_{12}q}}{2a_{11}(1+q)}, \tag{9}$$

$$y = q\frac{a_{11} \pm \sqrt{a_{11}^2(1+q)^2 - 4a_{11}(1+q)a_{12}q}}{2a_{11}(1+q)}, \tag{10}$$

$$z = 1 - (1+q)\frac{a_{11} \pm \sqrt{a_{11}^2(1+q)^2 - 4a_{11}(1+q)a_{12}q}}{2a_{11}(1+q)}\Big), m = 3.$$

Proof. To see that the vertices of the simplex in $\mathbb{R}_3$ are equilibrium points, from (14Theoretical resultsequation.5.14) we have

$$\begin{aligned} a_{12}f_2(\cdot)x_ty_t &= a_{11}x_tf_1(\cdot)(1-x_t-y_t), \\ a_{21}f_3(\cdot)x_ty_t &= a_{22}y_tf_4(\cdot)(1-x_t-y_t). \end{aligned} \tag{11}$$

By inspection, in each of the vertices $(x=1, y=0, z=0)$, $(x=0, y=1, z=0)$, and $(x=0, y=0, z=1)$, the above set of equations are satisfied and both left- and right-hand side are null.

We prove now that there may exist a fourth equilibrium point of type $(x, qx, 1-(1+q)x)$. To show this, from (16Theoretical resultsequation.5.16) we have

$$y_t = \frac{a_{11}f_1(\cdot)a_{21}f_3(\cdot)}{a_{12}f_2(\cdot)a_{22}f_4(\cdot)}x_t. \tag{12}$$

The underlying idea is that each consensus dynamics satisfies the invariance property

$$\frac{f_1(\cdot)f_3(\cdot)}{f_2(\cdot)f_4(\cdot)} = \begin{cases} 1 & \text{Case 1, 4 and 5,} \\ \frac{x^{m-1}}{y^{m-1}} & \text{Case 2 and 3.} \end{cases} \tag{13}$$

From the above we obtain

$$q = \begin{cases} \frac{a_{11}a_{21}}{a_{12}a_{22}} & \text{Case 1, 4 and 5,} \\ \sqrt[m]{\frac{a_{11}a_{21}}{a_{12}a_{22}}} & \text{Case 2 and 3.} \end{cases} \tag{14}$$

Therefore we may have a fourth equilibrium point characterised by

$$\begin{cases} y = \frac{a_{11}a_{21}}{a_{12}a_{22}}x & \text{Case 1, 4 and 5,} \\ y = \sqrt[m]{\frac{a_{11}a_{21}}{a_{12}a_{22}}}x & \text{Case 2 and 3.} \end{cases} \tag{15}$$

To prove (1) let us recall that for Case 1 we have $f_1(\cdot) = f_2(\cdot) = f_3(\cdot) = f_4(\cdot) = 1$ and therefore the equilibrium conditions (15Theoretical resultsequation.5.15) can be rewritten as

$$\begin{aligned} a_{12}y &= a_{11}(1-x-y), \\ a_{21}x &= a_{22}(1-x-y). \end{aligned} \tag{16}$$

From $y = qx = \frac{a_{11}a_{21}}{a_{12}a_{22}}x$, in the first equation of (16) one obtains $x = \frac{a_{11}}{a_{12}q+a_{11}(1+q)}$ from which we have (1).

Let us now prove (3). To this purpose, let us recall that in Case 4 we have $f_1(\cdot) = f_4(\cdot) = 1$ and $f_2(\cdot) = f_3(\cdot) = \frac{1}{x+y}$ and therefore the equilibrium conditions (15Theoretical resultsequation.5.15) can be rewritten as:

$$\begin{aligned} a_{12}y &= a_{11}(x+y)(1-x-y), \\ a_{21}x &= a_{22}(x+y)(1-x-y). \end{aligned} \tag{17}$$

From $y = qx$, the first equation of (17) is equivalently written as $a_{12}q = -a_{11}(1+q^2)x + a_{11}(1+q)$ from which we obtain $x = \frac{a_{11}(1+q)-a_{12}q}{a_{11}(1+q^2)}$ which in turn implies (3).

To prove (5), let us consider Case 5 which is characterised by $f_1(\cdot) = f_4(\cdot) = \frac{1}{x+y}$ and $f_2(\cdot) = f_3(\cdot) = 1$. The equilibrium conditions (15Theoretical resultsequation.5.15) yield

$$\begin{aligned} a_{12}y(x+y) &= a_{11}(1-x-y), \\ a_{21}x(x+y) &= a_{22}(1-x-y). \end{aligned} \tag{18}$$

Using $y = qx$, the first equation of (18) becomes $a_{21}(1+q)x^2 + a_{22}(1+q)x - a_{22} = 0$ from which only one root is nonnegative and yields (5).

To show that (6) and (7) are true, let us consider Case 2 and the corresponding functions $f_1(\cdot) = f_4(\cdot) = 1$ and $f_2(\cdot) = y^{m-1}$ and $f_3(\cdot) = x^{m-1}$. The equilibrium conditions (15Theoretical resultsequation.5.15) yield

$$\begin{aligned} a_{12}y^m = & \quad a_{11}(1-x-y), \\ a_{21}x^m = & \quad a_{22}(1-x-y). \end{aligned} \tag{19}$$

From $y = qx$, the first equation of (19) yields $a_{21}q^m x^m + a_{11}(1+q)x - a_{11} = 0$. For $m = 2$ the last equation becomes $a_{21}q^2x^2 + a_{11}(1+q)x - a_{11} = 0$ whose only nonnegative root is as in (6). For large m the first term $a_{21}q^m x^m$ can be approximated by a sufficiently small scalar ϵ_m and therefore the equation simplifies as $\epsilon_m + a_{11}(1+q)x - a_{11} = 0$ from which we get the approximation in (7).

Let us now show that (8) and (10) are true. To do this, let us recall that in Case 3 we have $f_1(\cdot) = x^{m-1}$, $f_2(\cdot) = f_3(\cdot) = 1$ and $f_4(\cdot) = y^{m-1}$. From the equilibrium conditions (15Theoretical resultsequation.5.15) we obtain

$$\begin{aligned} a_{12}y = & \quad a_{11}x^{m-1}(1-x-y), \\ a_{21}x = & \quad a_{22}y^{m-1}(1-x-y). \end{aligned} \tag{20}$$

From $y = qx$, the first equation of (20) yields $a_{11}(1+q)x^{m-1} - a_{11}x^{m-2} + a_{12}q = 0$. For $m = 2$ the above equation becomes $a_{11}(1+q)x - a_{11} + a_{12}q = 0$ which yields (8). For $m = 3$ we get $a_{11}(1+q)x^2 - a_{11}x + a_{12}q = 0$ whose roots are as in (10). $\square$
