# Supplementary material for: An evolutionary game perspective on quantised consensus in opinion dynamics
Source: PLoS One. 2019 Jan 4;14(1):e0209212. doi: 10.1371/journal.pone.0209212 (PMC6319711; doi:10.1371/journal.pone.0209212)
Supplement: S4 File — (PDF) [file pone.0209212.s004.pdf]

S4 File Corollary for the case where $q \simeq 1$

Corollary 1 (Symmetric case, $q \simeq 1$)**.** *Below is the list of equilibrium points $y = qx$ and corresponding feasibility conditions in the symmetric case where $q \approx 1$*

$$\textit{(Case 1)}\Big(x = \frac{a_{11}}{a_{12}+2a_{11}},\ y = \frac{a_{11}}{a_{12}+2a_{11}},\ z = 1-2\frac{a_{11}}{a_{12}+2a_{11}}\Big), \tag{1}$$

$$\textit{(Case 4)}\Big(x = \frac{2a_{11}-a_{12}}{2a_{11}},\ y = \frac{2a_{11}-a_{12}}{2a_{11}},\ z = 1-2\frac{2a_{11}-a_{12}}{2a_{11}}\Big), \quad a_{11} \le a_{12}, \tag{2}$$

$$\textit{(Case 5)}\Big(x = \frac{-a_{11}+\sqrt{a_{11}^2+2a_{12}a_{11}}}{2a_{12}},\ y = \frac{-a_{11}+\sqrt{a_{11}^2+2a_{12}a_{11}}}{2a_{12}}, \tag{3}$$
$$z = 1-2\frac{-a_{11}+\sqrt{a_{11}^2+2a_{12}a_{11}}}{2a_{12}}\Big), \quad \sqrt{a_{11}^2+2a_{12}a_{11}} \le 2a_{11},$$

$$\textit{(Case 2)}\Big(x = \frac{-a_{11}+\sqrt{a_{11}^2+a_{12}a_{11}}}{a_{12}},\ y = \frac{-a_{11}+\sqrt{a_{11}^2+a_{12}a_{11}}}{a_{12}}, \tag{4}$$
$$z = 1-2\frac{-a_{11}+\sqrt{a_{11}^2+a_{12}a_{11}}}{a_{12}}\Big),\ m = 2, \quad \frac{-a_{11}+\sqrt{a_{11}^2+a_{12}a_{11}}}{a_{12}} \le \frac{1}{2},$$

$$\Big(x = \frac{1}{2},\ y = \frac{1}{2},\ z = 0\Big),\ m\ \textit{large}, \tag{5}$$

$$\textit{(Case 3)}\Big(x = \frac{a_{11}-a_{12}}{2a_{11}},\ y = \frac{a_{11}-a_{12}}{2a_{11}},\ z = 1-\frac{a_{11}-a_{12}}{a_{11}}\Big), m = 2, \quad \frac{a_{11}-a_{12}}{a_{11}} \le 1, \tag{6}$$

$$\Big(x = \frac{1}{4} \pm \frac{a_{11}^2-2a_{11}a_{12}}{2a_{11}},\ y = \frac{1}{4} \pm \frac{a_{11}^2-2a_{11}a_{12}}{2a_{11}}, \tag{7}$$
$$z = 1-\frac{1}{4} \pm \frac{a_{11}^2-2a_{11}a_{12}}{a_{11}}\Big), m = 3, \quad 0 \le \frac{1}{4} \pm \frac{a_{11}^2-2a_{11}a_{12}}{a_{11}} \le 1.$$

Proof. To prove (1)-(7) it suffices to set $q = 1$ in the equilibrium equations of Theorem 2. The feasibility conditions are obtained by imposing that $0 \le x = y \le \frac{1}{2}$. □
