# Supplementary material for: An evolutionary game perspective on quantised consensus in opinion dynamics
Source: PLoS One. 2019 Jan 4;14(1):e0209212. doi: 10.1371/journal.pone.0209212 (PMC6319711; doi:10.1371/journal.pone.0209212)
Supplement: S5 File — (PDF) [file pone.0209212.s005.pdf]

S5 File Proof of theorem 3.

Stability of equilibrium points Let us rewrite (8) of the manuscript in matrix form as:

$$\begin{bmatrix} x_{t+1} \\ y_{t+1} \end{bmatrix} = \begin{bmatrix} 1 - a_{12}f_2(\cdot) - a_{11}f_1(\cdot) + a_{11}\partial_x f_1(\cdot)(1-x-y) & -a_{12}x\partial_y f_2(\cdot) - a_{11}f_1(\cdot) \\ -a_{21}y\partial_x f_4(\cdot) - a_{22}f_3(\cdot) & 1 - a_{21}f_4(\cdot) - a_{22}f_3(\cdot) + a_{22}\partial_y f_3(\cdot)(1-x-y) \end{bmatrix} \begin{bmatrix} x_t \\ y_t \end{bmatrix}. \tag{1}$$

A way to study the stability of the equilibrium solutions is to study the eigenvalues of the Jacobian matrix J of the non-linear state space model (1) evaluated in each equilibrium point. Let $\lambda = \{\lambda_1, \lambda_2\}$ be the eigenvalues of the Jacobian. If for a specific solution $|\lambda_i| < 1, \forall i \in \{1,2\}$ this solution is stable. We are ready to establish the following stability properties.

Theorem. *The following is a list of stable and unstable equilibrium points for each case.*

(Case 1) $\Big(x=1,\, y=0,\, z=0\Big), \Big(x=0,\, y=1,\, z=0\Big)$ *stable*
$\Big(x=0,\, y=0,\, z=1\Big), (x, qx, 1-(1+q)x)$ *unstable*

(Case 2) $\Big(x=1,\, y=0,\, z=0\Big), \Big(x=0,\, y=1,\, z=0\Big)$, *stable*
$(x, qx, 1-(1+q)x)$ *stable if*
$\mathcal{A} - \mathcal{B} < x < \mathcal{A} + \mathcal{B} \bigwedge \Big(q \le \frac{4-a_{11}}{a_{22}} \bigvee \Big(\frac{4-a_{11}}{a_{22}} < q < \frac{4}{a_{22}} \bigwedge x < \frac{4-a_{22}}{a_{11}}\Big)\Big)$,
where $\mathcal{A} = \frac{3m^2+q+3m^2q}{2m^2(2+5q+2q^2)}$, $\mathcal{B} = \frac{1}{2}\sqrt{\frac{m^4+6m^2q-2m^4q+q^2+6m^2q^2+m^4q^2}{m^4(2+5q+2q^2)^2}}$

$\Big(x=0,\, y=0,\, z=1\Big)$ *unstable*

(Case 3) $\Big(x=1,\, y=0,\, z=0\Big), \Big(x=0,\, y=1,\, z=0\Big), \Big(x=0,\, y=0,\, z=1\Big)$ *stable*
$(x, qx, 1-(1+q)x)$ *unstable*

(Case 4) $\Big(x=1,\, y=0,\, z=0\Big), \Big(x=0,\, y=1,\, z=0\Big)$ *stable*
$\Big(x=0,\, y=0,\, z=1\Big), (x, qx, 1-(1+q)x)$ *unstable*

(Case 5) $\Big(x=1,\, y=0,\, z=0\Big), \Big(x=0,\, y=1,\, z=0\Big)$, *stable* $\Big(x=$
$(x, qx, 1-(1+q)x)$ *unstable*

Proof. . In Case 1, the Jacobian of (1) is given by:

$$J = \begin{bmatrix} 1 - a_{12}y - a_{11}x + a_{11}(1-x-y) & -a_{12}x - a_{11}x \\ -a_{21}y - a_{22}y & 1 - a_{21}x - a_{22}y + a_{22}(1-x-y) \end{bmatrix}. \tag{2}$$

The vertices $(x=1, y=0, z=0), (x=0, y=1, z=0)$ are asymptotically stable since the eigenvalues of the Jacobian matrix evaluated in these points are smaller than one. In particular, their eigenvalues are $(\lambda_1 = 1 - a_{11}, \lambda_2 = 1 - a_{21})$ and $(\lambda_1 = 1 - a_{22}, \lambda_2 = 1 - a_{12})$ respectively which are always between zero and one. The vertex $x = 0, y = 0, z = 1$ is unstable since both its eigenvalues are greater than one. The second

eigenvalue of the Jacobian evaluated in $y = qx$ is always greater than one and therefore it is an unstable solution. The Jacobian evaluated in $y = qx$ and its corresponding eigenvalues are in (S6 Appendix.).

Let us now investigate Case 2. The Jacobian of (1) is obtained as:

$$J = \begin{bmatrix} 1 - a_{12}y^m - a_{11}x + a_{11}(1-x-y) & -a_{12}my^{m-1}x - a_{11}x \\ -a_{21}myx^{m-1} - a_{22}y & 1 - a_{21}x^m - a_{22}y + a_{22}(1-x-y) \end{bmatrix}. \quad (3)$$

To see that the vertices $(x = 1, y = 0, z = 0), (x = 0, y = 1, z = 0)$ are asymptotically stable, note that the corresponding eigenvalues of the Jacobian evaluated in the vertices are $(\lambda_1 = 1 - a_{11}, \lambda_2 = 1 - a_{21})$ and $(\lambda_1 = 1 - a_{22}, \lambda_2 = 1 - a_{12})$, respectively. The vertex $x = 0, y = 0, z = 1$ is unstable since both its eigenvalues are greater than one.

An equivalent set of rules, instead of studying the eigenvalues are the following: $\left|\frac{Tr(J)}{2}\right| < 1 \bigwedge 1 + \Delta(J) > Tr(J)$, where $Tr(J)$ is the trace of the Jacobian matrix J and $\Delta(J)$ is its determinant.The trace of the Jacobian estimated in the equilibrium satisfied the aforementioned rule if either $q \leq \frac{4-a_{11}}{a_{22}}$ or if $\frac{4-a_{11}}{a_{22}} < q < \frac{4}{a_{22}} \bigwedge x < \frac{4-a_{22}}{a_{11}}$. The expression $1+\Delta(J) > Tr(J)$ is satisfied when $\mathcal{A} - \mathcal{B} < x < \mathcal{A} + \mathcal{B}$. The Jacobian evaluated in $y = qx$ and its corresponding eigenvalues, trace and determinant are in (S6 Appendix.).

As for Case 3, the Jacobian of (1) is obtained as

$$J = \begin{bmatrix} 1 - a_{12}y + ma_{12}y - \frac{a_{12}xy}{1-x-y} & -a_{12}x - \frac{a_{12}xy}{1-x-y} \\ -a_{21}y - \frac{a_{21}xy}{1-x-y} & 1 - a_{21}x + ma_{21}x - \frac{a_{21}xy}{1-x-y} \end{bmatrix}. \quad (4)$$

The vertices $(x = 1, y = 0, z = 0), (x = 0, y = 1, z = 0)$ are asymptotically stable. Indeed, the corresponding eigenvalues of the Jacobian evaluated in the vertices are $(\lambda_1 = 1 - a_{11}, \lambda_2 = 1 - a_{21})$ and $(\lambda_1 = 1 - a_{12}, \lambda_2 = 1 - a_{22})$ respectively. The vertex $x = 0, y = 0, z = 1$ is stable since both its eigenvalues are equal to one. The equilibria which correspond to $y = qx$ are unstable. The Jacobian evaluated in $y = qx$ and its corresponding eigenvalues, determinant and trace are presented in (S6 Appendix.).

As regards Case 4, we obtain for the Jacobian of (1) as follows:

$$J = \begin{bmatrix} 1 - a_{12}y\frac{y^2}{(x+y)^2} - a_{11}x + a_{11}(1-x-y) & -a_{12}x\frac{x^2}{(x+y)^2} - a_{11}x \\ -a_{21}y\frac{y^2}{(x+y)^2} - a_{22}y & 1 - a_{21}x\frac{x^2}{(x+y)^2} - a_{22}y + a_{22}(1-x-y) \end{bmatrix}. \quad (5)$$

The vertices $(x = 1, y = 0, z = 0), (x = 0, y = 1, z = 0)$ are asymptotically stable. The corresponding eigenvalues of the Jacobian evaluated in the vertices are $(\lambda_1 = 1 - a_{11}, \lambda_2 = 1 - a_{21})$ and $(\lambda_1 = 1 - a_{22}, \lambda_2 = 1 - a_{12})$ respectively. The vertex $x = 0, y = 0, z = 1$ is unstable since its eigenvalues are greater than one. The rules, $\left|\frac{Tr(J)}{2}\right| < 1 \wedge 1 + \Delta(J) > Tr(J)$, are not satisfied together when the Jacobian is evaluated in $y = qx$. The Jacobian evaluated in $y = qx$ and its corresponding eigenvalues, trace and determinant are in (S6 Appendix.).

Finally, for Case 5, the Jacobian of (1) is:

$$J = \begin{bmatrix} 1 - a_{12}y - a_{11}\frac{x}{x+y} + a_{11}(1-x-y)\frac{y^2}{(x+y)^2} & -a_{12}x - a_{11}\frac{x}{x+y} - a_{11}\frac{x}{(x+y)^2}(1-x-y) \\ -a_{21}x - a_{22}\frac{y}{x+y} - a_{22}\frac{x}{(x+y)^2}(1-x-y) & 1 - a_{21}x - a_{22}\frac{y}{x+y} + a_{22}(1-x-y)\frac{x^2}{(x+y)^2} \end{bmatrix} \quad (6)$$

The vertices $(x = 1, y = 0, z = 0), (x = 0, y = 1, z = 0)$ are asymptotically stable. The corresponding eigenvalues of the Jacobian evaluated in the

vertices are ($\lambda_1 = 1 - a_{11}, \lambda_2 = 1 - a_{21}$) and ($\lambda_1 = 1 - a_{22}, \lambda_2 = 1 - a_{12}$) respectively. The vertex $x = 0, y = 0, z = 1$ is also stable since both its eigenvalues are equal to one. The rules, $\left|\frac{Tr(J)}{2}\right| < 1 \wedge 1 + \Delta(J) > Tr(J)$, are not satisfied together when the Jacobian is evaluated in $y = qx$. The Jacobian, evaluated in $y = qx$, and its corresponding eigenvalues, trace and determinant are in (S6 Appendix.). $\square$
