# Supplementary material for: An evolutionary game perspective on quantised consensus in opinion dynamics
Source: PLoS One. 2019 Jan 4;14(1):e0209212. doi: 10.1371/journal.pone.0209212 (PMC6319711; doi:10.1371/journal.pone.0209212)
Supplement: S6 File — (PDF) [file pone.0209212.s006.pdf]

S6 File Appendix

Jacobian and Eigenvalues of the Markov process resulted from linear cross inhibition signal The Jacobian of the Markov chain resulted from:

$$A = \begin{array}{c} \\ X \\ Y \\ Z \end{array} \begin{array}{c} \begin{array}{ccc} X & Y & Z \end{array} \\ \begin{pmatrix} a_{11} & -a_{12} & 0 \\ -a_{21} & a_{22} & 0 \\ 0 & 0 & 0 \end{pmatrix} \end{array}, \tag{1}$$

evaluated in $y = qx$ is:

$$J = \begin{pmatrix} \frac{a_{12}(a_{21}+a_{22})+a_{11}(a_{21}-a_{12}a_{22})}{(a_{11}a_{21}+a_{12}(a_{21}+a_{22}))} & -\frac{a_{12}(a_{11}+a_{12})a_{22}}{(a_{11}a_{21}+a_{12}(a_{21}+a_{22}))} \\ -\frac{a_{11}a_{21}(a_{21}+a_{22})}{(a_{11}a_{21}+a_{12}(a_{21}+a_{22}))} & \frac{a_{12}(a_{21}+a_{22})+a_{11}(a_{21}-a_{21}a_{22})}{(a_{11}a_{21}+a_{12}(a_{21}+a_{22}))} \end{pmatrix}.$$

The corresponding eigenvalues are:

$$\lambda_1 = -\frac{1}{2(a_{11}a_{21}+a_{12}(a_{21}+a_{22}))}\Big(a_{11}a_{21}(-2+a_{22}) + a_{11}a_{12}a_{22} - 2a_{12}(a_{21}+a_{22}) + \sqrt{a_{11}}\sqrt{a_{22}}\sqrt{4a_{12}^2 a_{21}(a_{21}+a_{22}) + a_{11}(a_{12}^2 a_{22} + a_{21}^2 a_{22} + 2a_{12}a_{21}(2a_{21}+a_{22}))}\Big),$$

$$\lambda_2 = \frac{1}{2(a_{11}a_{21}+a_{12}(a_{21}+a_{22}))}\Big(2a_{12}(a_{21}+a_{22}) - a_{11}(a_{21}(-2+a_{22}) + a_{12}a_{22}) + \sqrt{a_{11}}\sqrt{a_{22}}\sqrt{4a_{12}^2 a_{21}(a_{21}+a_{22}) + a_{11}(a_{12}^2 a_{22} + a_{21}^2 a_{22} + 2a_{12}a_{21}(2a_{21}+a_{22}))}\Big).$$

The absolute value of the second eigenvalue is always greater than 1, $|\lambda_2| > 1$, and therefore the equilibrium is unstable.

Jacobian and Eigenvalues of the Markov process resulted from the case of weak cross inhibitory signal The Jacobian of the Markov chain resulted from:

$$A(x,y) = \begin{array}{c} \\ X \\ Y \\ Z \end{array} \begin{array}{c} \begin{array}{ccc} X & Y & Z \end{array} \\ \begin{pmatrix} a_{11} & -a_{12}y^{m-1} & 0 \\ -a_{21}x^{m-1} & a_{22} & 0 \\ 0 & 0 & 0 \end{pmatrix} \end{array}. \tag{2}$$

evaluated in $y = qx$ is:

$$J = \begin{pmatrix} 1 - a_{11}x & a_{11}x\left(1 - m\frac{1}{qx}(1-x-qx)\right) \\ a_{22}qx\left(1 - m\frac{1}{x}(1-x-qx)\right) & 1 - a_{22}qx \end{pmatrix}.$$

The corresponding eigenvalues are:

$$\lambda_1 = \tfrac{1}{2q^2x^2}\Big(2q^2x^2 - a_{11}q^2x^3 - a_{22}q^3x^3 - q^{\frac{3}{2}}x^2\Big(4a_{11}a_{22}m^2 - 8a_{11}a_{22}m^2x + 8a_{11}a_{22}mqx - 8a_{11}a_{22}m^2qx + 4a_{11}a_{22}m^2x^2 + a_{11}^2qx^2 - 8a_{11}a_{22}mqx^2 + 8a_{11}a_{22}m^2qx^2 + 2a_{11}a_{22}q^2x^2 - 8a_{11}a_{22}mq^2x^2 + 4a_{11}a_{22}m^2q^2x^2 + a_{22}^2q^3x^2\Big)^{\frac{1}{2}}\Big),$$

$$\lambda_2 = \frac{1}{2q^2x^2}\Bigg(2q^2x^2 - a_{11}q^2x^3 - a_{22}q^3x^3 + q^{\frac{3}{2}}x^2\Big(4a_{11}a_{22}m^2 - 8a_{11}a_{22}m^2x + 8a_{11}a_{22}mqx- 8a_{11}a_{22}m^2qx + 4a_{11}a_{22}m^2x^2 + a_{11}^2qx^2 - 8a_{11}a_{22}mqx^2 + 8a_{11}a_{22}m^2qx^2 + 2a_{11}a_{22}q^2x^2- 8a_{11}a_{22}mq^2x^2 + 4a_{11}a_{22}m^2q^2x^2 + a_{22}^2q^3x^2\Big)^{\frac{1}{2}}\Bigg).$$

The trace and the determinant of the Jacobian are respectively:

$$2 - a_{11}x - a_{22}qx$$

and

$$1 - \frac{a_{11}a_{22}m^2}{q} - a_{11}x - 2a_{11}a_{22}mx + 2a_{11}a_{22}m^2x + \frac{2a_{11}a_{22}m^2x}{q} -a_{22}qx + 2a_{11}a_{22}mx^2 - 2a_{11}a_{22}m^2x^2 - \frac{a_{11}a_{22}m^2x^2}{q} + 2a_{11}a_{22}mqx^2 - a_{11}a_{22}m^2qx^2.$$

Jacobian and Eigenvalues of the Markov process resulted from waggle dance signal The Jacobian of the Markov chain resulted from:

$$A(x,y) = \begin{array}{c} \\ X \\ Y \\ Z \end{array}\begin{array}{c} \begin{array}{ccc} X & Y & Z \end{array} \\ \begin{pmatrix} a_{11}x^{m-1} & -a_{12} & 0 \\ -a_{21} & a_{22}y^{m-1} & 0 \\ 0 & 0 & 0 \end{pmatrix} \end{array}, \tag{3}$$

evaluated in $y = qx$ is:

$$J = \begin{pmatrix} 1 + a_{12}qx\left(m - 1 - \frac{x}{1-x-qx}\right) & -a_{12}x\left(1 + \frac{x}{1-x-qx}\right) \\ a_{21}qx\left(1 + \frac{x}{1-x-qx}\right) & 1 + a_{21}x\left(m - 1 - \frac{qx}{1-x-qx}\right) \end{pmatrix}.$$

The corresponding eigenvalues are:

$$\lambda_1 = \frac{1}{2(-1+x+qx)}\left(-2 + 2x + a_{21}x - a_{21}mx + 2qx + a_{12}qx - a_{12}mqx - a_{21}x^2 + a_{21}mx^2+ a_{12}mqx^2 + a_{21}mqx^2 - a_{12}q^2x^2 + a_{12}mq^2x^2 - (x^2(a_{21}^2(1 - x + m(-1 + x + qx))^2+ a_{12}^2q^2(1 - qx + m(-1 + x + qx))^2 - 2a_{12}a_{21}q(-1 + (-1 + 3q)x+ (q - 2q^2)x^2 + m^2(-1 + x + qx)^2 - m(2 - 3(1 + q)x + (1 + q)^2x^2)))^{\frac{1}{2}}\right),$$

$$\lambda_2 = \frac{1}{2(-1+x+qx)}\left(-2 + 2x + a_{21}x - a_{21}mx + 2qx + a_{12}qx - a_{12}mqx - a_{21}x^2 + a_{21}mx^2+ a_{12}mqx^2 + a_{21}mqx^2 - a_{12}q^2x^2 + a_{12}mq^2x^2 + (x^2(a_{21}^2(1 - x + m(-1 + x + qx))^2+ a_{12}^2q^2(1 - qx + m(-1 + x + qx))^2 - 2a_{12}a_{21}q(-1 + (-1 + 3q)x + (q - 2q^2)x^2+ m^2(-1 + x + qx)^2 - m(2 - 3(1 + q)x + (1 + q)^2x^2)))^{\frac{1}{2}}\right).$$

The trace and the determinant of the Jacobian are respectively:

$$2 + a_{12}qx(-1 + m - x/(1 - x - qx)) + a_{21}x(-1 + m - (qx)/(1 - x - qx)),$$

and

$$\frac{1}{(-1+x+qx)^2}(1 + (-2 + a_{21}(-1 + m) + (-2 + a_{12}(-1 + m))q)x+ (1 + (2 + a_{12} - 2a_{12}m)q + (1 + 2a_{12} - 2a_{12}m)q^2 + a_{21}(2 + q + a_{12}m^2q- 2m(1 + q + a_{12}q)))x^2 + (a_{12}q(1 + q)(m - q + mq) + a_{21}(-1 - (1 + a_{12})q + a_{12}q^2- 2a_{12}m^2q(1 + q) + m(1 + q)(1 + q + 3a_{12}q)))x^3 + a_{12}a_{21}q(-(-1 + q)q - m(1 + q)^2 +m^2(1 + q)^2)x^4).$$

Jacobian and Eigenvalues of the Markov process resulted from neighbor-based cross-inhibitory signal The Jacobian of the Markov chain resulted from:

$$A(x,y) = \begin{array}{c} \\ X \\ Y \\ Z \end{array} \begin{array}{c} \begin{array}{ccc} X & Y & Z \end{array} \\ \begin{pmatrix} a_{11} & -a_{12}\frac{1}{x+y} & 0 \\ -a_{21}\frac{1}{x+y} & a_{22} & 0 \\ 0 & 0 & 0 \end{pmatrix} \end{array}. \tag{4}$$

evaluated in $y = qx$ is:

$$J = \begin{pmatrix} 1 - a_{12}\frac{q^3}{(1-q)^2}x + a_{11}(1-x-qx) - a_{11}x & -a_{12}\frac{1}{(1+q)^2}x - a_{11}x \\ a_{21}\frac{q^3}{(1-q)^2}x - a_{22}qx & 1 - a_{21}\frac{1}{(1-q)^2}x + a_{22}(1-x-qx) + a_{22}qx \end{pmatrix}.$$

The corresponding eigenvalues are:

$$\begin{aligned}
\lambda_1 = {} & -\tfrac{1}{2(-1+q^2)^2)}(-2 + a_{11}x - a_{22} + 4q^2 + 2(-a_{11}x + a_{22})q^2 + (-2 + a_{11}x - a_{22})q^4 + \\
& (1+q)^2(a_{21} + a_{22} - 3a_{22}q^2 + (a_{12} + 2a_{22})q^3)x + a_{11}(-1+q^2)^2(-1+x+qx) + \\
& (((1+q)^2((a_{11}x + a_{22})^2(-1+q)^4(1+q)^2 - \\
& 2(a_{11}x + a_{22})(-1+q^2)^2(a_{21} - a_{12}q^3 + a_{22}(-1+q)^2(1+2q))x + \\
& (a_{21}^2(1+q)^2 + a_{12}^2q^6(1+q)^2 + a_{22}^2(-1+q)^4(1+q)^2(1+2q)^2 + 2a_{21}(a_{12}q^3(1+(-6+q)q) + \\
& a_{22}(1+2q)(-1+q^2)^2) - 2a_{12}a_{22}(-1+q)^2q(-2+q(4+q(-1+q(4+q(5+2q))))))x^2 + \\
& a_{11}^2(-1+q)^4(1+q)^2(-1+x+qx)^2 + 2a_{11}(a_{11}x(-1+q)^4(1+q)^2(-1+x+qx) + \\
& (-1+q^2)^2x(a_{21} + a_{21}(-1-q+2q^3)x + a_{12}q^3(-1+x+qx)) - \\
& a_{22}(-1+q)^4(1+q)^2(1+x(-2+x+q(-3+x+2qx)))))))^{\frac{1}{2}}),
\end{aligned}$$

$$\begin{aligned}
\lambda_2 = {} & \tfrac{1}{2(-1+q^2)^2)}(-2 + a_{11}x - a_{22} + 4q^2 + 2(-a_{11}x + a_{22})q^2 + (-2 + a_{11}x - a_{22})q^4 + \\
& (1+q)^2(a_{21} + a_{22} - 3a_{22}q^2 + (a_{12} + 2a_{22})q^3)x + a_{11}(-1+q^2)^2(-1+x+qx) + \\
& (((1+q)^2((a_{11}x + a_{22})^2(-1+q)^4(1+q)^2 \\
& -2(a_{11}x + a_{22})(-1+q^2)^2(a_{21} - a_{12}q^3 + a_{22}(-1+q)^2(1+2q))x + \\
& (a_{21}^2(1+q)^2 + a_{12}^2q^6(1+q)^2 + a_{22}^2(-1+q)^4(1+q)^2(1+2q)^2 + \\
& 2a_{21}(a_{12}q^3(1+(-6+q)q) + a_{22}(1+2q)(-1+q^2)^2) - \\
& 2a_{12}a_{22}(-1+q)^2q(-2+q(4+q(-1+q(4+q(5+2q))))))x^2 + \\
& a_{11}^2(-1+q)^4(1+q)^2(-1+x+qx)^2 + 2a_{11}(a_{11}x(-1+q)^4(1+q)^2(-1+x+qx) + \\
& (-1+q^2)^2x(a_{21} + a_{21}(-1-q+2q^3)x + a_{12}q^3(-1+x+qx)) - \\
& a_{22}(-1+q)^4(1+q)^2(1+x(-2+x+q(-3+x+2qx)))))))^{\frac{1}{2}}).
\end{aligned}$$

The trace and the determinant of the Jacobian are respectively:

$$2 - a_{11}x - (a_{21}x)/(1-q)^2 - a_{22}qx - (a_{12}q^3x)/(1-q)^2 + a_{11}(1-x-qx) + a_{22}(1-x-qx)$$

and

$$\begin{aligned}
& \tfrac{1}{(-1+q)^4(1+q)^2)}(-(-1+a_{11}x)(1+a_{22})(-1+q)^4(1+q)^2 + (-1+q^2)^2((-1+a_{11}x)(a_{21}+a_{22}) \\
& -3(-1+a_{11}x)a_{22}q^2 - (a_{12} + (2 - 2a_{11}x + a_{12})a_{22})q^3)x + a_{12}q(4a_{21}q^3 + \\
& a_{22}(-1+q)^2(-1+2q+q^3(4+q(5+2q))))x^2 + a_{11}((1+a_{22})(-1+q)^4(1+q)^2 - \\
& (-1+q^2)^2(a_{21} + (-1+q)^2(1+2a_{22}+q+3a_{22}q))x + (-1+q^2)^2(a_{21}(1+q-q^3) + \\
& a_{22}(-1+q)^2(1+2q(1+q)))x^2)).
\end{aligned}$$

Jacobian and Eigenvalues of the Markov process resulted from neighbor-based waggle dance signal The Jacobian of the Markov chain resulted from:

$$A(x,y) = \begin{array}{c} \\ X \\ Y \\ Z \end{array} \begin{array}{c} \begin{array}{ccc} X & Y & Z \end{array} \\ \begin{pmatrix} a_{11}\frac{1}{x+y} & -a_{12} & 0 \\ -a_{21} & a_{22}\frac{1}{x+y} & 0 \\ 0 & 0 & 0 \end{pmatrix} \end{array}. \tag{5}$$

evaluated in $y = qx$ is:

$$J = \begin{pmatrix} 1 + a_{12}qx + a_{11}(1-x-qx)\frac{q^2}{(1-q)^2} - a_{11}\frac{q}{1-q} & -a_{12}x - a_{11}\frac{1}{1+q} \\ -a_{21}qx - a_{22}\frac{q}{1+q} & 1 - a_{21}x + a_{22}(1-x-qx) - a_{22}\frac{q}{1+q} \end{pmatrix}.$$

The corresponding eigenvalues are:

$$\lambda_1 = -(1/(2(1+q)^2))(-2 + a_{11} - a_{22} - 4q + a_{11}q + a_{22}q - 2q^2 - a_{11}q^2 + a_{22}q^2 + a_{11}q^2qx+ \\ a_{21}x + a_{22}x + a_{12}qx + 2a_{21}qx + a_{22}qx + a_{11}q^2x + 2a_{12}q^2x + a_{21}q^2x+ \\ a_{12}q^3x(((a_{22}(-1 + q + q^2 + x + qx) + (1 + q)^2(-2 + a_{21}x + a_{12}qx)+ \\ a_{11}(1 + q + q^2(-1 + qx + x)))^2 - 4((1 + q)^4(1 - a_{12}qx + a_{21}x(-1 - a_{12}xq + a_{12}qx))+ \\ a_{22}(1 + q)^2(1 - x + a_{12}q^3x - q(1 + a_{12}x + x + a_{12}x - a_{12}x^2) + q^2(-1 - a_{12}x + a_{12}x(1 + x))) \\ +a_{11}(a_{22}(-1 + x + q^4(-1 + qx + x) + q^3(1 + x)(-1 + qx + x) + q(-1 + 2x)+ \\ q^2(1 + qx(-1 + x) - x + x^2)) + (1 + q)^2(-1 - q + a21x+ \\ q^2(1 - x - 2a_{21}x + a_{21}x^2 + qx(-1 + a_{21}x)))))))^{\frac{1}{2}}),$$

$$\lambda_2 = -(1/(2(1+q)^2))(-2 + a_{11} - a_{22} - 4q + a_{11}q + a_{22}q - 2q^2 - a_{11}q^2 + a_{22}q^2 + a_{11}q^2qx+ \\ a_{21}x + a_{22}x + a_{12}qx + 2a_{21}qx + a_{22}qx + a_{11}q^2x + 2a_{12}q^2x + a_{21}q^2x+ \\ a_{12}q^3x - (((a_{22}(-1 + q + q^2 + x + qx) + (1 + q)^2(-2 + a_{21}x + a_{12}qx)+ \\ a_{11}(1 + q + q^2(-1 + qx + x)))^2 - 4((1 + q)^4(1 - a_{12}qx + a_{21}x(-1 - a_{12}xq + a_{12}qx)) \\ +a_{22}(1 + q)^2(1 - x + a_{12}q^3x - q(1 + a_{12}x + x + a_{12}x - a_{12}x^2) + q^2(-1 - a_{12}x + a_{12}x(1 + x))) \\ +a_{11}(a_{22}(-1 + x + q^4(-1 + qx + x) + q^3(1 + x)(-1 + qx + x) + q(-1 + 2x)+ \\ q^2(1 + qx(-1 + x) - x + x^2)) + (1 + q)^2(-1 - q + a_{21}x+ \\ q^2(1 - x - 2a_{21}x + a_{21}x^2 + qx(-1 + a_{21}x)))))))^{\frac{1}{2}}).$$

The trace and the determinant of the Jacobian are respectively:

$$2-a_{11}/(1+q)-(a_{22}q)/(1+q)+(a_{11}q^2(1-qx-x))/(1+q)^2-a_{21}x-a_{12}qx+(a_{22}(1-x-qx))/(1+q)^2$$

and

$$1 - a_{12}qx + a_{21}x(-1 - a_{12}xq + a_{12}qx) + (a_{22}(1 - x + a_{12}q^3x - q(1 + a_{12}x + x + a_{12}x - a_{12}x^2) \\ +q^2(-1 - a_{12}x + a_{12}x(1 + x))))/(1 + q)^2 + (1/((1 + q)^4))a_{11}(a_{22}(-1 + x + q^4(-1 + qx + x)+ \\ q^3(1 + x)(-1 + qx + x) + q(-1 + 2x) + q^2(1 + qx(-1 + x) - x + x^2)) \\ +(1 + q)^2(-1 - q + a_{21}x + q^2(1 - x - 2a_{21}x + a_{21}x^2 + qx(-1 + a_{21}x)))).$$
